# Flame Retardancy and Hydrophobicity Enhancement for HDPE Containing an Organic–Inorganic Hybrid P–N–Si–Cu Cooperative Flame-Retardant System

**DOI:** 10.3390/polym18212707

**Published:** 2026-11-01

**Authors:** Yan Li, Ming Yan, Yuyao Zhong, Xuheng Chen, Tianshuo Zhang, Yue Gao, Fan Yang

**Affiliations:** 1 School of Electrical and Electronic Engineering, North China Electric Power University, Baoding 071003, China; 13043150082@163.com (M.Y.); 18741293688@163.com (Y.Z.); 19131963566@163.com (X.C.); 15033225745@163.com (T.Z.); 13514429446@163.com (Y.G.); y19588573832@163.com (F.Y.); 2 Hebei Key Laboratory of Green and Efficient New Electrical Materials and Equipment, Baoding 071003, China

**Keywords:** high-density polyethylene, siloxane network, lignin-based organic skeleton

## Abstract

High-density polyethylene (HDPE) has been widely used as packaging and protection materials owing to its electrical insulation and mechanical strength. However, HDPE is flammable and requires flame-retardant modification. The addition of flame retardant leads to deteriorated compatibility of blends, reducing hydrophobic performance. Balancing the flame retardancy and hydrophobicity of the blend is challenging. Ammonium polyphosphate (APP) was modified by sequential loading of Cu-MOF followed by silane coupling agent treatment to prepare Si-Cu-APP, and lignin (Lig) was modified via piperazine-formaldehyde to obtain lignin-based organic skeleton (Pip-Lig). The Si-Cu-APP/Pip-Lig system achieves P–N–Si–Cu quaternary cooperative flame-retardant effect. while this system reduces the intramolecular hydroxyl content and weakens the polarity of the system. It makes the surface energy of the blend lower and improves its internal dispersion, forming an efficient hydrophobic protective layer. At a total additive loading of 30 wt%, the blend exhibits an LOI of 28.5%. The peak heat release rate (PHRR) decreases by 68.45%, and the total smoke production (TSP) decreases by 26.52%. Its water contact angle rises from 91.58° to 124.24°, water absorption decreases by 72%, and flame-retardant leaching is only 0.11%. This work provides an effective strategy for fabricating HDPE with simultaneously enhanced flame retardancy and hydrophobicity.

## 1. Introduction

High-density polyethylene (HDPE) is widely used in packaging, construction, transportation and electrical fields owing to its, excellent electrical and mechanical properties [1,2,3,4]. However, the molecular structure of HDPE consists entirely of carbon-hydrogen bonds, with a limiting oxygen index (LOI) of only 17–18%, classifying it as a highly flammable material [5]. It releases dense smoke during combustion accompanied by severe molten dripping, which not only fails to achieve self-extinguishing but also ignites adjacent materials via the dripping products, significantly increasing fire hazards [6]. Therefore, it is necessary to conduct research on high-efficiency flame-retardant modification of HDPE.

While improving the flame retardancy of HDPE, the enhancement of its hydrophobic performance also deserves considerable attention. If the material has insufficient surface hydrophobicity, moisture can easily infiltrate and permeate into the material matrix through the surface under long-term humid conditions, accelerating the hydrolysis and migration of polar flame retardants [7]. It impairs the stability of the flame-retardant system, leading to a decreased performance [8]. In electrical application scenarios, surface hydrophilicity and moisture absorption will increase the leakage current, reduce the insulation performance of the material, and pose potential safety hazards [9]. Therefore, hydrophobic modification is an essential prerequisite for ensuring the long-term stable service of HDPE blends under humid working conditions.

In the early stage, halogenated flame retardants were used for flame-retardant HDPE owing to their high flame-retardant efficiency. However, they tended to release toxic and corrosive gases during combustion, posing potential hazards to both human health and the ecological environment [10]. Therefore, they are gradually being replaced by halogen-free flame-retardant systems. Conventional inorganic halogen-free flame retardants, such as magnesium hydroxide [11], aluminum hydroxide [12] have the merits of non-toxicity and low cost. However, to achieve desirable flame-retardant efficiency, a high loading of more than 50 wt% is generally required, which significantly deteriorates the mechanical properties and processing fluidity of the matrix [13]. In comparison, phosphorus-nitrogen flame-retardant systems can form a dense intumescent char layer upon combustion [14], which may effectively blocks heat and oxygen transfer, making it one of the most extensively studied strategies for high-efficiency halogen-free flame retardancy. However, most intumescent systems rely on petroleum-based raw materials and exhibit limited biodegradability, which makes it difficult to meet the requirements of sustainable development [15].

Against this background, there has been a growing body of research on lignin (Lig) used for flame retardants. Lig contains a large number of hydroxyl and methoxy functional groups in its structure [16], which endows it with exceptional carbonization potential. Weng et al. [17] grafted P and N elements onto Lig and applied it as a flame retardant in phenolic foams. The blends exhibited significantly enhanced flame retardancy and smoke suppression properties. Liang et al. [18] grafted ammonium polyphosphate (APP) and melamine onto Lig through a one-step reaction to make the flame retardant for epoxy resin (EP). Guo et al. [19] grafted P onto Lig using phosphoric acid and urea. The incorporation of the flame retardant imparted excellent flame retardancy and char-forming ability to PAN. As can be seen from the above, Lig can be used as a flame retardant to improve the flame-retardant properties of polymers. Nevertheless, when Lig is used alone in hydrocarbon polymers (e.g., polyethylene), its flame-retardant effect is limited. In general, it needs to be combined with other components to achieve better performance. Although its aromatic structure provides a carbon-rich framework for char formation, Lig alone may not generate a sufficiently effective protective char layer to adequately inhibit heat transfer, oxygen diffusion, and the release of combustible volatiles. Therefore, combining Lig with complementary flame-retardant components is a promising strategy for enhancing its overall flame-retardant effectiveness [20].

As an acid source component, APP can form a dense and intumescent char layer through catalytic dehydration and carbonization [21]. After compounding with modified Lig, the flame retardancy of the blends can be significantly improved [22]. However, both APP and Lig contain abundant hydroxyl groups in their molecular structures [23,24], resulting in strong polarity. They tend to form hydrogen bonds with water molecules and thus exhibit high hydrophilicity, which may lead to water absorption and performance degradation in humid service environments. Therefore, the hydrophobic performance of the system deserves attention, whereas systematic studies in this area are still insufficient.

In recent years, biomimetic organic-inorganic hybrid design has become an effective means to improve the multi-functional properties of biomass-derived polymers via rational combination of biomass-based organic matrices and inorganic functional components. Liu et al. [25] fabricated a biomimetic periosteum by incorporating calcium phosphate nanoparticles into GelMA hydrogel electrospun fibers, and this organic-inorganic hybrid system achieved simultaneous improvement in osteogenic and angiogenic performances. From a more general perspective, Ouyang et al. [26] proposed an integrated “interface-dynamic crosslinking-hierarchical-structure” design framework for biomimetic hybrid soy-protein bio-adhesives. They demonstrated that appropriate organic-inorganic hybridization can synergistically optimize multiple properties of bio-based materials, including mechanical strength, toughness, water resistance and mildew resistance. Inspired by the above-mentioned studies, introducing functional inorganic components into the modified lignin matrix is expected to simultaneously enhance the flame-retardant and hydrophobic properties of composite materials.

In addition to selecting appropriate phosphorus- and nitrogen-containing components, the introduction of metal-containing species represents a potential strategy to tailor the charring process and the protective performance of the resulting carbonaceous residues. Among diverse metal–organic frameworks (MOFs), Copper-based Metal–Organic Framework(Cu-MOF) are attractive because the redox activity and thermal transformation behavior of copper-containing species may affect the catalytic charring process and the formation of protective char layers [27]. Moreover, when copper-containing species are combined with APP, they can interact with phosphorus-containing thermal decomposition products, which enables the integration of metal-assisted charring and APP-induced dehydrative charring. Previous investigations on Cu-MOF/APP systems have verified the feasibility of this strategy for improving the flame retardancy of polymer composites [28].

**Figure 1:**
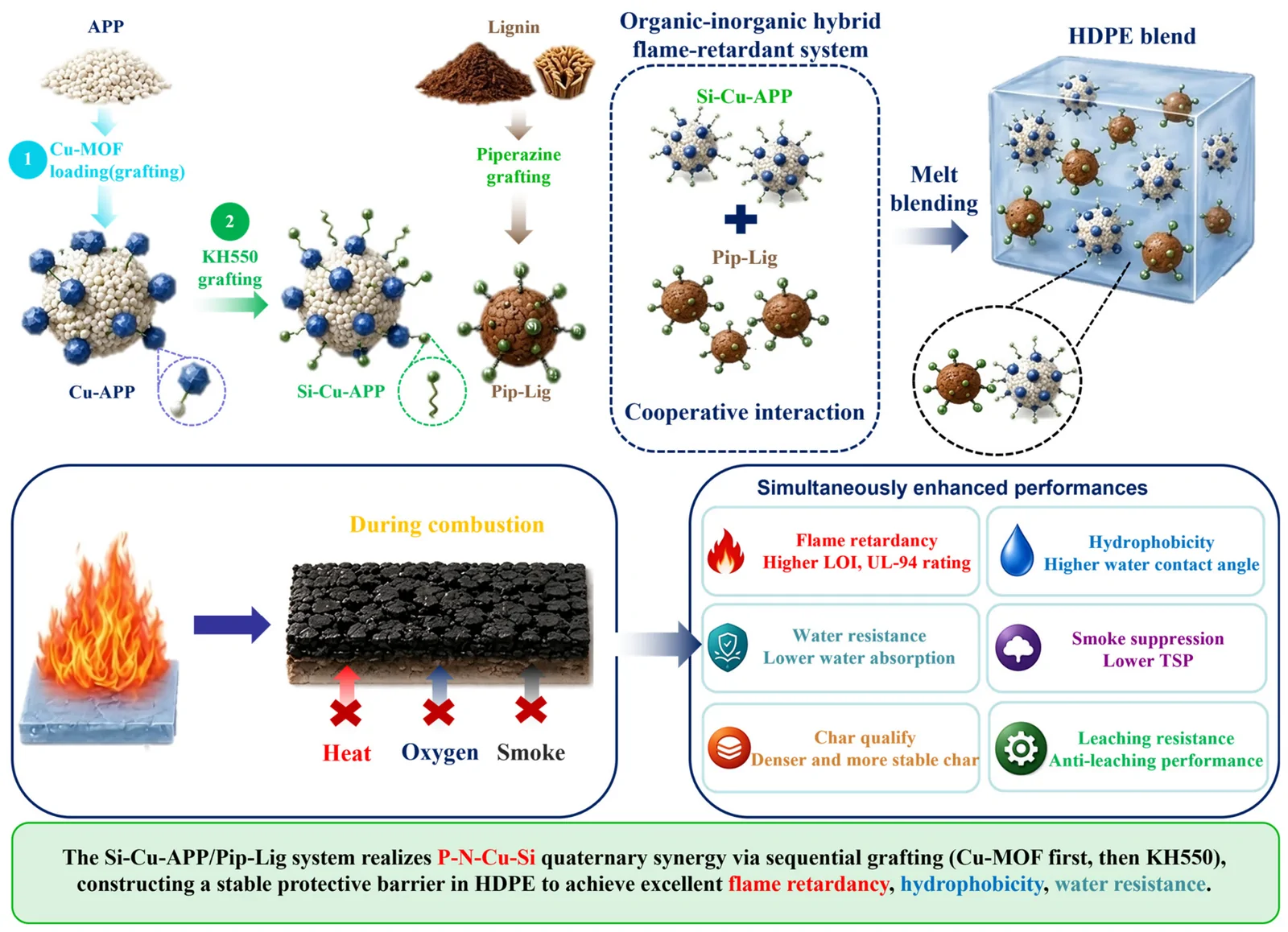
Schematic diagram of HDPE blends with high hydrophobicity and flame retardancy achieved via cooperative effect of Si-Cu-APP and Pip-Lig.

In this study, Cu-MOF and silane coupling agent (KH550) modified APP was compounded with a novel nitrogen-grafted Lig fabricated via nucleophilic substitution and polycondensation. The two components construct an organic–inorganic hybrid flame-retardant system. The phosphorus-containing acidic components are produced by the thermal decomposition of Si-Cu-APP. These components act cooperatively with the aromatic skeleton and nitrogen-containing active groups of Pip-Lig. Meanwhile, this structure significantly decreases intramolecular hydroxyl content and reduces the polarity of the system. Consequently, the blends possess lower surface energy and better internal dispersion. A highly hydrophobic protective layer is formed. They are incorporated into HDPE through melt blending, as illustrated in Figure 1. Characterizations of the flame retardancy and hydrophobicity of the resulting blends verified that the introduction of this flame-retardant system not only enhanced the flame resistance of HDPE but also improved its hydrophobicity, thereby prolonging the service life of the flame retardant.

## 2. Experimental Section

### 2.1. Materials

APP, piperazine, formaldehyde solution, glacial acetic acid, and γ-aminopropyltriethoxysilane (KH550) were sourced from Shanghai Macklin Biochemical Technology Co., Ltd. Alkali Lig was provided by TCI (Shanghai) Development Co., Ltd. HDPE was obtained from Dongguan Jinheng Plastic Co., Ltd. in China. Anhydrous ethanol was purchased from Kermel Chemical Reagent Co., Ltd. in China. Potassium hydroxide solution was supplied by Shenzhen Bolinda Technology Co., Ltd. in China. Copper (II) nitrate trihydrate (Cu(NO_3_)_2_·3H_2_O), terephthalic acid (H_2_BDC), N, N-dimethylformamide (DMF), and triethylamine were purchased from Aladdin Biochemical Technology Co., Ltd., China.

### 2.2. Synthesis of Modified APP

Initially, 5 g of APP was dispersed in 100 mL anhydrous ethanol and treated by ultrasonication for 30 min, followed by suction filtration. The sample was vacuum-dried at 60 °C for 12 h to complete the pretreatment of APP. Subsequently, 0.6 g Cu(NO_3_)_2_·3H_2_O and 0.4 g H_2_BDC were dissolved in 30 mL and 20 mL DMF, respectively. After complete dissolution, the two solutions were mixed and stirred for 15 min. Afterwards, 5 g pretreated APP was added into the mixed solution, and the mixture was reacted at 50 °C for 2 h. Then, 0.2 mL triethylamine was slowly dropwise added to promote the in-situ crystallization and growth of Cu-MOF on the surface of APP. Upon the completion of the reaction, the product was separated by centrifugation and washed three times with anhydrous ethanol. Finally, the precipitate was vacuum-dried at 60 °C for 12 h to obtain copper-modified APP intermediate (Cu-APP).

Following this, 2 g of KH550 was dissolved in 200 mL of 95% ethanol aqueous solution. After stirring at room temperature for 10 min, 0.5 mL of glacial acetic acid was added dropwise to adjust the pH to 4–5, and the solution was stirred for another 30 min to prepare a stable silanol solution. After that, 20 g of Cu-APP was added into the solution. The mixture was agitated at 500 r/min. The solution was heated to 70 °C and held at this temperature for 5 h. After the reaction, the mixture was cooled to room temperature naturally. 5 wt% ammonia water was added dropwise to adjust the pH to 7–8, and the solution was stirred at room temperature for 1 h. The product was centrifuged and washed with ethanol solution three times to remove unreacted reagents and impurities. Ultimately, the product was vacuum-dried at 60 °C for 12 h to obtain KH550 and Cu-MOF cooperativeally modified APP (Si-Cu-APP).

### 2.3. Synthesis of Modified Lig

Firstly, 300 mL of 4 wt% potassium hydroxide solution was poured into a three-necked flask equipped with a magnetic stirrer, followed by the slow addition of 30 g of Alkali Lig. After heating the mixture to 60 °C, 12.9 g of anhydrous piperazine was added into the reaction system in three equal portions. The amount of 24 mL of 37 wt% formaldehyde solution was added dropwise to the mixed solution in four equal portions, and the pH of the system was strictly controlled at 9.0–9.5 throughout the process. After the dropwise addition of formaldehyde was completed, the temperature of the constant temperature water bath was slowly raised to 75 °C, and the reaction was continued with mechanical stirring for 3 h. The reaction solution was cooled to below 40 °C, and 10% (*v*/*v*) glacial acetic acid solution was slowly added dropwise under stirring to adjust the pH to 5–6. The precipitate was collected by vacuum filtration and washed alternately with deionized water and anhydrous ethanol for three times in succession. The solid product was placed in a vacuum drying oven and dried at 70 °C for 12 h to obtain tan piperazine-formaldehyde modified Lig powder (Pip-Lig).

### 2.4. Preparation of HDPE Blends

The HDPE blends were prepared via melt blending. Table 1 lists the corresponding formulations. Prior to use, APP, Si-Cu-APP, Pip-Lig and Lig were fully dried in a vacuum oven. HDPE and the relevant flame retardants were carefully weighed according to the required ratios and mixed in a high-speed mixer for 10 min. The mixture was then melt-blended on a two-roll mill at temperatures of 175 °C with a roll speed of 40 rpm for 1 h. After blending, the material was hot-pressed at 175 °C and 20 MPa for 30 min to prepare test specimens.

### 2.5. Characterization

#### 2.5.1. Microscopic Characterization

Fourier transform infrared spectroscopy (FTIR) was performed on a Nicolet iS50 spectrometer from Thermo Fisher Scientific according to GB/T 6040-2019. X-ray photoelectron spectroscopy (XPS) was conducted using a Thermo Scientific ESCALAB 250Xi instrument. Scanning electron microscopy and energy-dispersive X-ray spectrometer (SEM/EDS) was done by the JSM-6390 LV scanning electron microscope in the Japan following GB/T 17359-2012.

#### 2.5.2. Flame Retardant Characterization

Thermogravimetric analysis (TGA) was performed on a PerkinElmer-STA6000 (USA) instrument under a nitrogen atmosphere at a heating rate of 10 °C/min over a temperature range of 30–800 °C in accordance with ISO 11358-1:2014. According to the ASTM D 2863 standard, LOI was measured using a LOI analyzer (JF-3A), with specimen dimensions of 150 × 6.5 × 3 mm^3^.In accordance with the UL-94 standard (ASTM D 3801), a horizontal-vertical flame chamber (CZF-3) was used to test specimens measuring 125 × 13 × 3 mm^3^.Based on the ISO 5660 standard, combustion performance was evaluated using a cone calorimeter (CCT, Fire Testing Technology, UK) at a heat flux of 50 kW/m^2^ for specimens of 100 × 100 × 3 mm^3^. Each test on the sample was repeated three times, and the experimental data were expressed as mean ± standard deviation. Thermogravimetric-Fourier transform infrared spectroscopy (TG-FTIR) was conducted using a combination of a TG 209 F1 (Netzsch, Germany) and a 170 SX FTIR spectrometer (Nicolet, USA) referring to ISO 11358-3:2018.

#### 2.5.3. Hydrophobicity Characterization

Contact angle measurements were carried out using a OCA20 instrument manufactured by Data Physics Instruments GmbH (Germany) following GB/T 30693-2014. Disc-shaped samples and 3 μL deionized water droplets were used for the tests, with a water droplet observation and recording time of 10 s.

The water resistance and anti-extraction properties were evaluated according to GB/T 11547–2008. Specimens (60 × 60 ± 1.0 mm, 1.0 ± 0.1 mm thick) were boiled in 500 mL deionized water at 70 °C for 168 h. Three replicates per formulation were analyzed gravimetrically for extraction rate (Equation (1)), and water absorption rate (Equation (2)).

Extraction rate = (m_1_ − m_3_)/m_1_ (1)

Water absorption rate = (m_2_ − m_3_)/m_1_ (2)

where m_1_ is initial mass before water immersion (g), m_2_ is mass after water immersion and surface drying (g), m_3_ is the mass after drying to constant weight post-immersion (g).

## 3. Results and Discussion

### 3.1. Characterization of Modified APP

**Figure 2:**
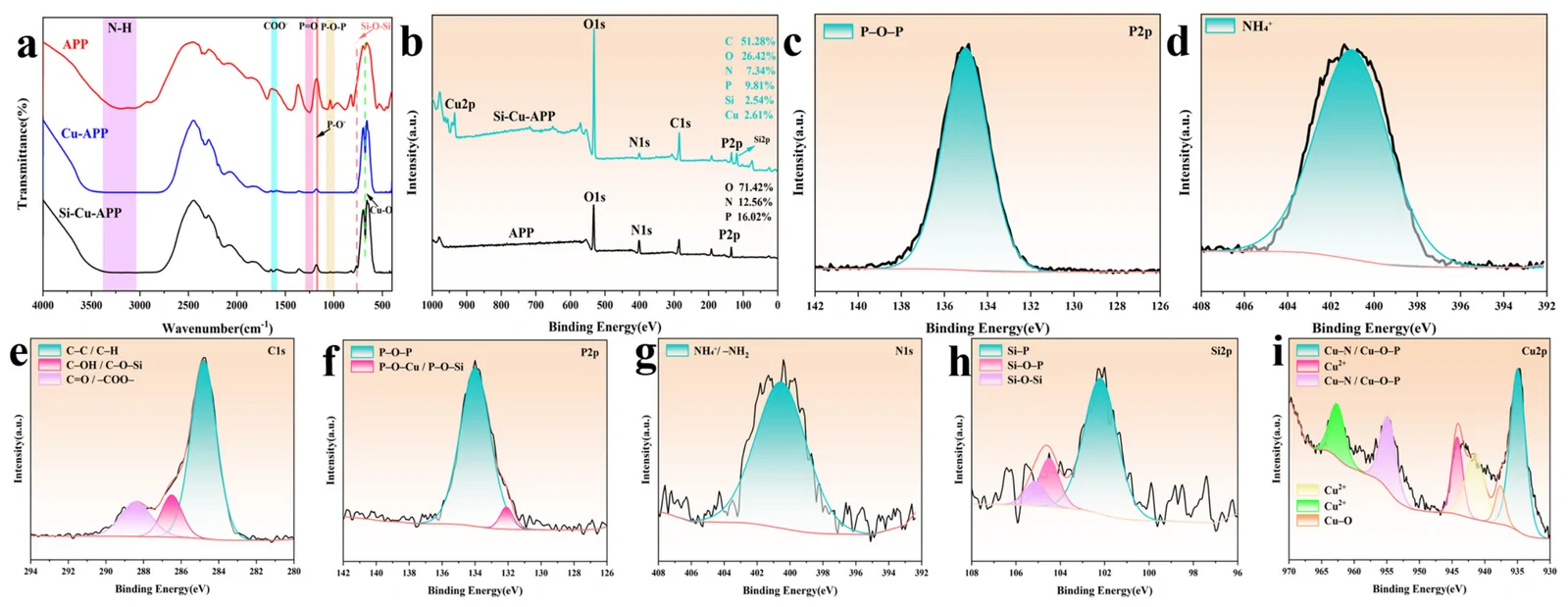
(**a**) FTIR spectra of APP, Cu-APP and Si-Cu-APP; (**b**) Survey XPS spectra of APP and Si-Cu-APP; (**c**,**d**) P2p, N1s fine XPS spectra of APP; (**e**–**i**) C1s, P2p, N1s, Si2p, Cu2p fine XPS spectra of Si-Cu-APP.

Figure 2 systematically characterized the chemical structure, surface elemental composition and bonding states of APP, Cu-APP and Si-Cu-APP by means of FTIR and XPS. Figure 2a shows the FTIR spectra of APP, Cu-APP and Si-Cu-APP. The peak of Si-Cu-APP at 3400–3100 cm^−1^ corresponds to the asymmetric stretching vibration of N–H [29]. A distinct P=O stretching vibration peak appears at 1260–1240 cm^−1^, and the characteristic stretching vibration peak of the P–O–P bond is observed in the range of 1100–1000 cm^−1^ [30]. Meanwhile, P–O^−^ vibration peak can be seen near 900 cm^−1^. These characteristic peaks collectively confirm that surface modification treatment does not change the intrinsic internal ammonium salt crystal structure of APP. The absorption band in the 1100–1000 cm^−1^ region is significantly broadened and intensified, which is mainly attributed to the superposition of the P–O–P vibration with the vibrations of Si–O–P bonds formed during the hydrolysis and condensation of KH550 [31]. In addition, a new absorption peak appears at 820 cm^−1^, which is mainly assigned to the characteristic vibration of copper-based coordination structures, accompanied by the overlapping vibrational signal of Si–O–Si bonds. It directly confirms that copper-based components and KH550 are jointly grafted onto the APP surface.

Figure 2b presented the survey XPS spectra of APP and Si-Cu-APP. As shown in Figure 2b, compared with APP, characteristic Cu2p and Si2p signals can be clearly detected for Si-Cu-APP, proving that copper-based components and silicon species have been successfully immobilized on the surface of APP. Figure 2c,d and Figure 2f,g displayed the high-resolution P2p and N1s spectra of pristine APP and Si-Cu-APP, respectively. For pristine APP, the P2p peak centered at 133.6 eV originates from P–O–P structures, and the N1s peak located at 401.0 eV corresponds to NH_4_^+^ within APP crystal. After surface modification, the P2p spectrum of Si-Cu-APP (Figure 2f) can be fitted into two peaks: the dominant peak at 133.4 eV belongs to P–O–P bonds, while the newly generated peak at 135.0 eV is attributed to P–O–Si/P–O–Cu bonds [32]. In the N1s spectrum of Si-Cu-APP (Figure 2g), apart from the characteristic peak of NH_4_^+^, an emerging peak at 400.1 eV is attributed to amine nitrogen (–NH_2_) derived from KH550 [33]. Figure 2e displays the high-resolution C1s spectrum of Si-Cu-APP, which can be split into three fitting peaks. The peak at 284.8 eV is assigned to C–C/C–H, the peak at 286.2 eV corresponds to C–OH/C–O–Si, and the peak at 288.5 eV belongs to C=O/COO– groups. The presence of C–O–Si signal further confirms the successful grafting of silane molecular chains. For the Si2p spectrum (Figure 2h), it can be deconvoluted into multiple characteristic peaks: the peak at 102.1 eV is assigned to Si–O–C bond, and the peak at 103.5 eV corresponds to Si–O–P/Si–O–Si bonds, demonstrating that hydrolyzed silane undergoes condensation reaction with P–OH groups on the APP surface to form stable Si–O–P covalent bonds. Moreover, the Cu2p spectrum (Figure 2i) further verifies the existence of copper-containing species on the particle surface. XPS analysis results demonstrate that modification with KH550 silane and copper-based coordination structures successfully introduces silicon and copper elements onto the surface of APP particles, which is consistent with the aforementioned FTIR results.

**Figure 3:**
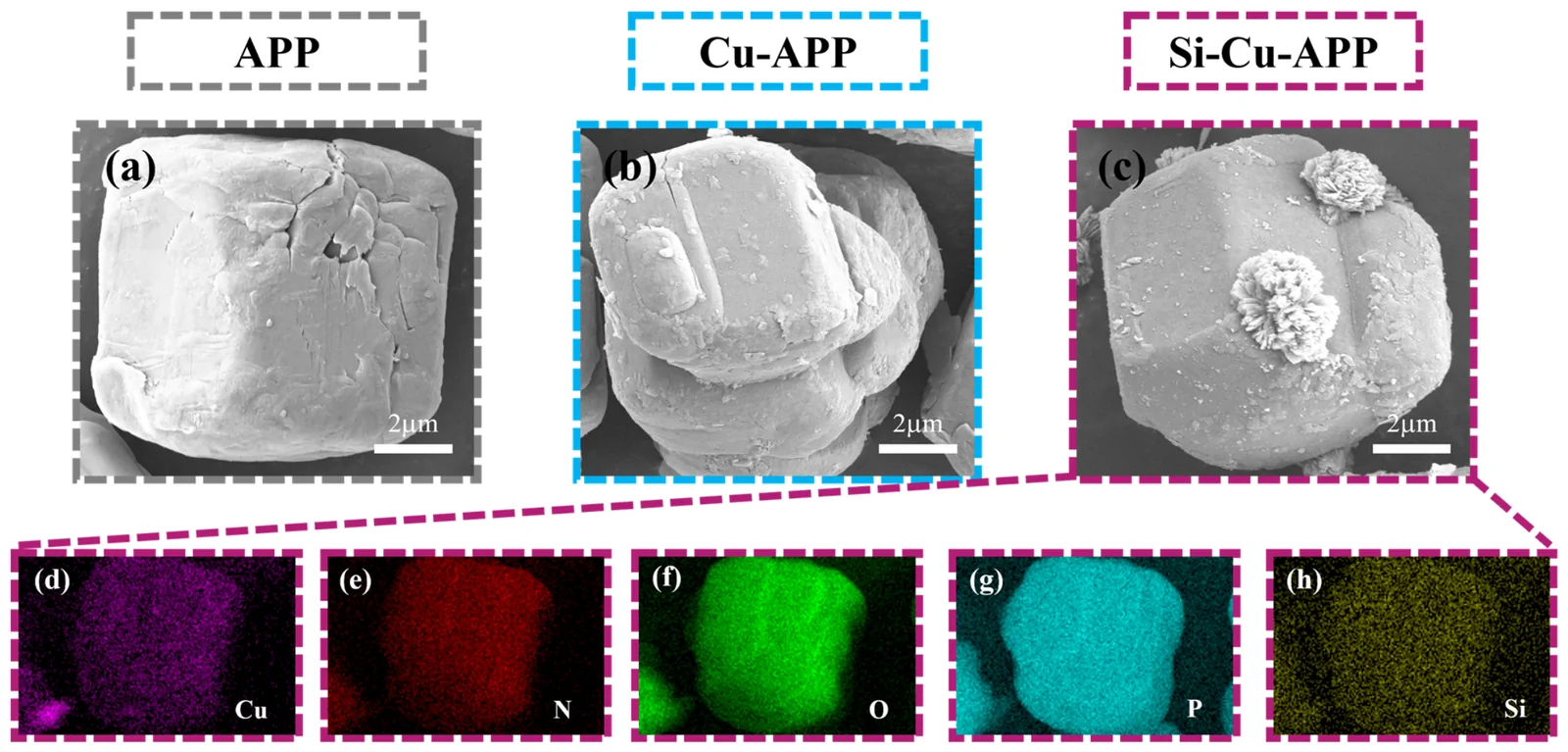
(**a**–**c**) SEM morphologies of APP, Cu-APP and Si-Cu-APP; (**d**–**h**) elemental mapping patterns of Cu, N, O, P and Si for Si-Cu-APP.

Figure 3a–c show the SEM images of APP, Cu-APP and Si-Cu-APP. As can be seen in Figure 3a, APP exhibits a regular blocky crystalline morphology with relatively smooth particle surfaces. Such smooth surface structure leads to weak interfacial interaction between APP and the HDPE matrix. Untreated APP particles tend to agglomerate during melt processing, which hinders the uniform dispersion of fillers within the matrix. After Cu-APP was prepared by in-situ loading of copper-based functional components on APP (Figure 3b), a small number of fine particulate precipitates could be observed on the crystal surface. Cu-APP was further modified via silanization with KH550 to obtain Si-Cu-APP (Figure 3c), on which abundant well-developed flower-like microstructures were formed, greatly increasing the surface roughness of particles. This effectively improves the interfacial compatibility between fillers and the HDPE matrix, and provides favorable conditions for the enhancement of flame retardancy and hydrophobicity of the blends. Elemental mapping results of Si-Cu-APP (Figure 3d–h) reveal that Cu, N, O, P and Si elements are homogeneously distributed over the particles, which directly confirms that both copper-based components and silane species have been successfully modified onto the surface of APP. The uniform distribution of various functional components on the surface of fillers facilitates the Si–Cu cooperative catalytic charring effect during combustion.

### 3.2. Characterization of Modified Lig

**Figure 4:**
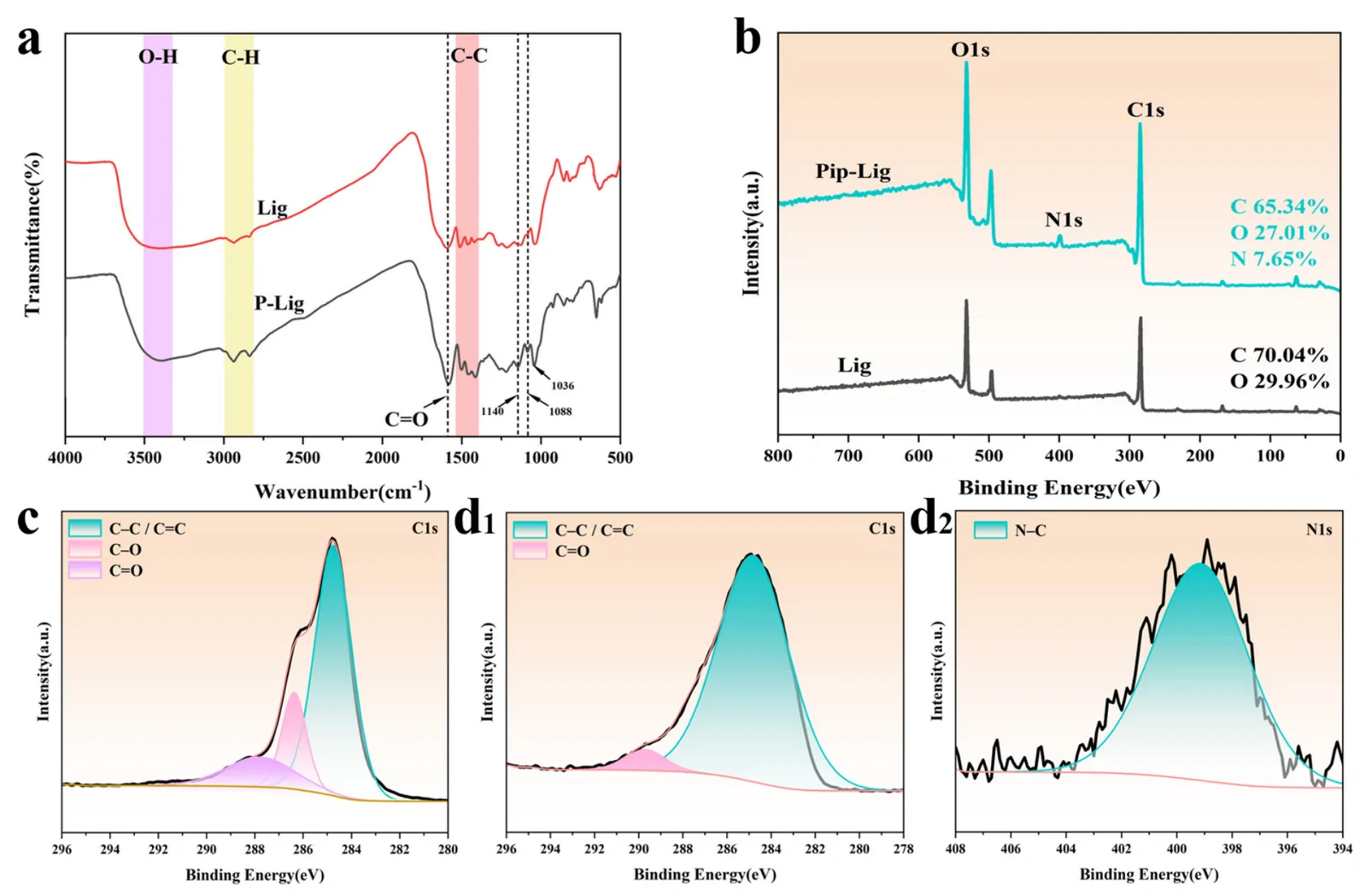
(**a**) FTIR and (**b**) XPS spectra of Lig, Pip-Lig; (**c**) C1s fine XPS spectrum of Lig; C1s, N1s fine XPS spectra for: (**d_1_**,**d_2_**) Pip-Lig.

Figure 4 systematically characterized the chemical structure, surface elemental composition and bonding states of Lig and Pip-Lig by means of FTIR and XPS. Figure 4a shows the FTIR spectra of Lig and Pip-Lig. As can be seen, the broad and strong absorption band at approximately 3400 cm^−1^ is mainly attributed to the stretching vibration of phenolic and aliphatic hydroxyl groups (–OH) in Lig [34]. The absorption peaks in the region of 2930–2850 cm^−1^ correspond to the stretching vibration of aliphatic C–H bonds. The characteristic absorption peaks at about 1600 cm^−1^ and 1510 cm^−1^ are assigned to the skeletal vibrations of the aromatic rings in Lig [35]. These characteristic peaks collectively confirm that Pip-Lig retains the backbone structure of Lig. Meanwhile, three additional distinct absorption peaks newly appeared or intensified at 1140 cm^−1^, 1088 cm^−1^ and 1036 cm^−1^, which are closely related to the stretching vibrations of C–N and C–O bonds [36]. These changes confirm that piperazine molecules have been successfully grafted onto the Lig structure via chemical bonding.

Figure 4b–d_2_ presented the XPS spectra of Lig and Pip-Lig. As shown in Figure 4b, compared with Lig, the nitrogen content in Pip-Lig was increased to 7.65%. This result directly confirms the successful introduction of nitrogen-containing functional groups. Figure 4c,d_1_ show the high-resolution C1s spectra of Lig and Pip-Lig, respectively. The peak at 284.8 eV corresponds to C–C/C=C bonds, 286.0 eV to C–O bonds, and 288.0 eV to C=O bonds. In contrast, the peak shape of Pip-Lig near 286 eV is broadened to some extent with a slight binding energy shift. This change can be attributed to the introduction of nitrogen-containing structures, where the −CH_2_−N− moieties formed via the Mannich reaction overlap with the original C–O chemical environment. Figure 4d_2_ shows the N1s spectrum of Pip-Lig, which can be fitted into one main characteristic peak at 399.8 eV, assigned to the –N–CH_2_– bond [37]. Detailed analysis of the N1s spectrum further verifies the successful introduction of nitrogen-containing functional groups, which is highly consistent with the conclusions obtained from the C1s spectrum and the aforementioned FTIR results. XPS analysis demonstrates that piperazine-formaldehyde modification successfully introduced nitrogen-containing functional groups onto the Lig molecular chains, thereby confirming the effectiveness of the modification process.

**Figure 5:**
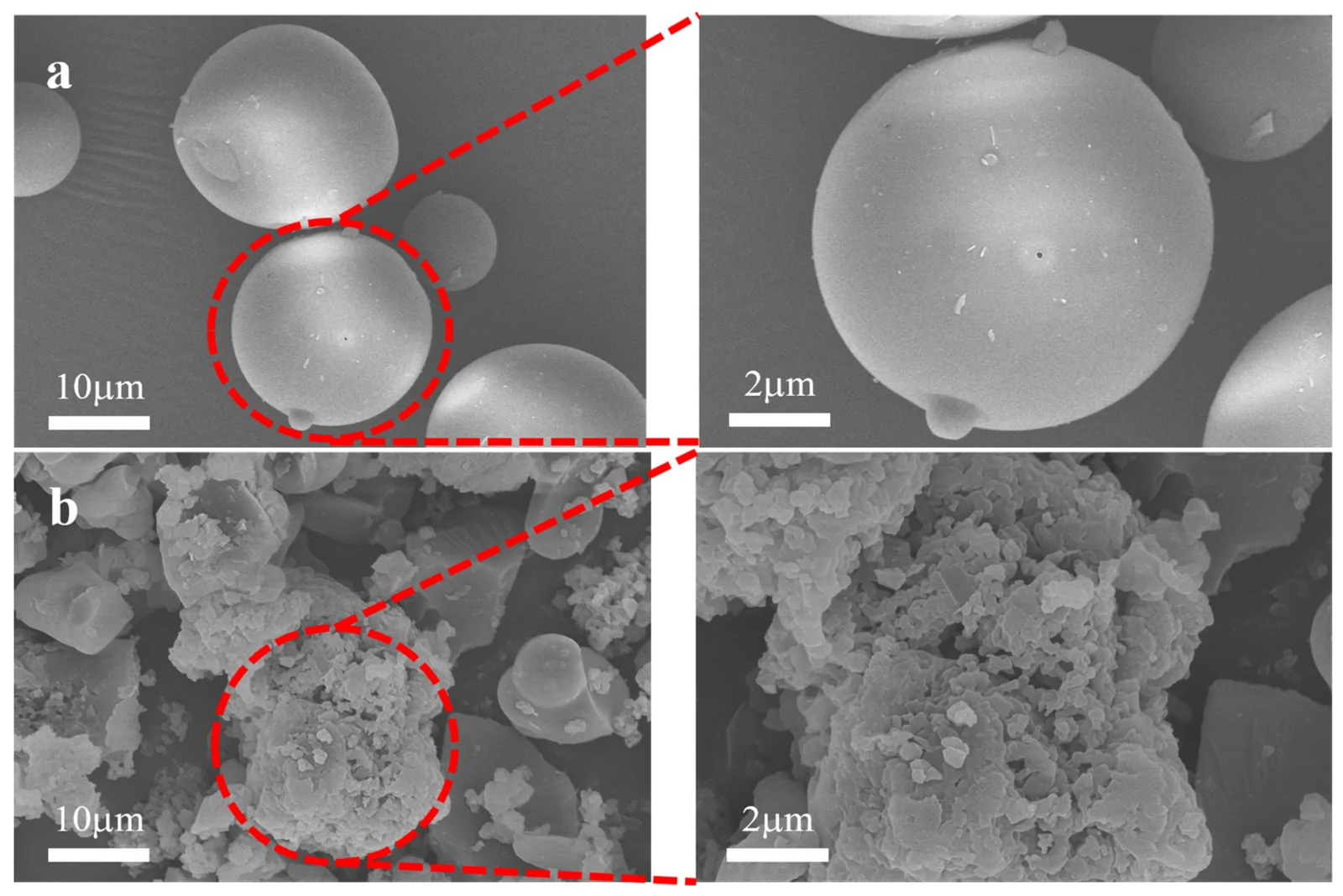
SEM images of (**a**) Lig and (**b**) Pip-Lig.

Figure 5 showed the SEM images of Lig and Pip-Lig. Lig has a relatively smooth surface mainly composed of polar groups such as hydroxyl groups, resulting in weak interfacial interaction with the HDPE matrix, easy agglomeration and poor dispersion. After piperazine-formaldehyde grafting modification, Pip-Lig exhibits unique wrinkled and roughened surface morphology with significantly increased specific surface area. This is because piperazine-formaldehyde is chemically grafted onto Lig, which disrupts the ordered structure of Lig. This structure not only strengthens the interaction with the HDPE matrix, but also provides efficient interfacial active sites for the subsequent cooperative effect with Si-Cu-APP.

**Figure 6:**
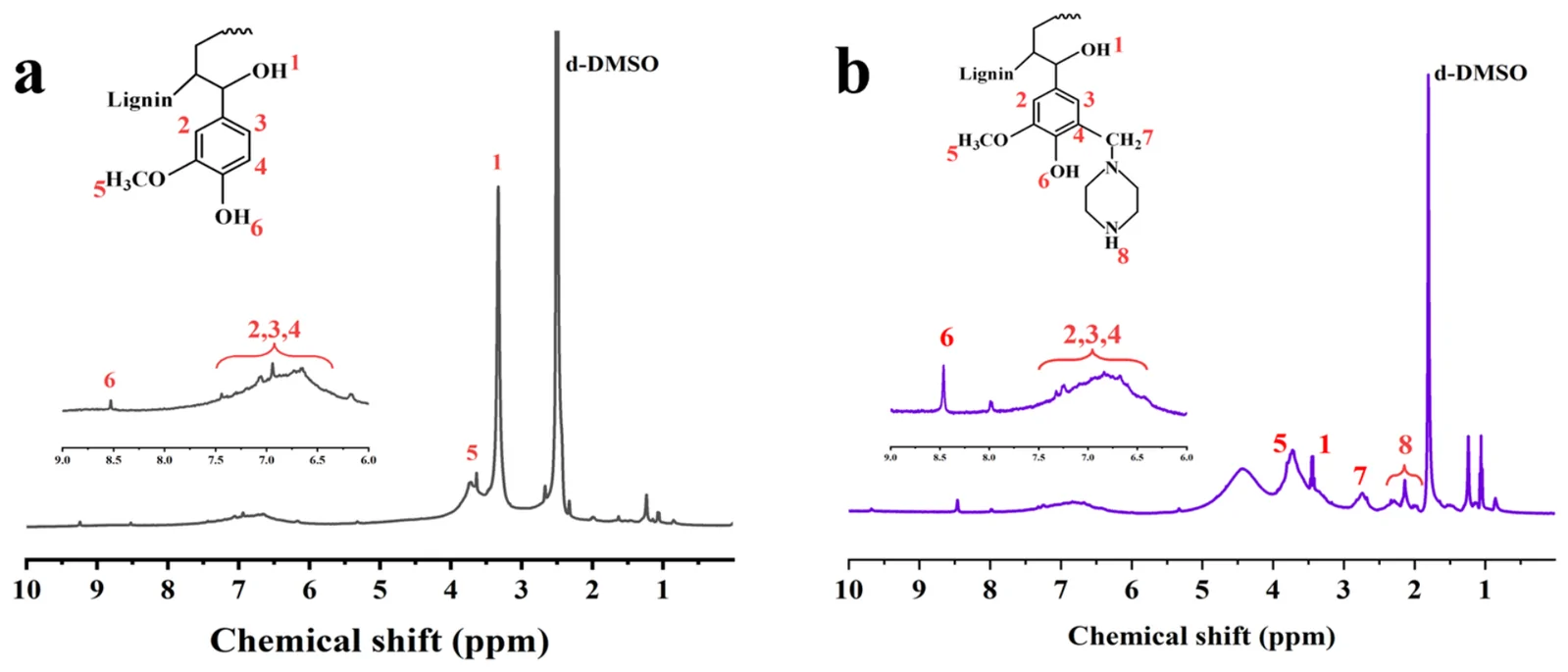
^1^H NMR spectra of (**a**)Lig and (**b**)Pip-Lig.

^1^H NMR is an efficient technique to investigate the chemical structures of Lig and Pip-Lig. Figure 6a presents the characteristic chemical shifts of Lig: the peak at 8.52 ppm is assigned to phenolic hydroxyl protons (H-6); the broad peaks ranging from 6.3 to 7.5 ppm correspond to aromatic protons (H-2, H-3 and H-4); the signal at 4.8 ppm belongs to aliphatic hydroxyl protons on the side chain (H-1), and the peak at 3.8 ppm is attributed to methoxy protons (H-5), which is consistent with previous literature [38]. The ^1^H NMR spectrum of Pip-Lig (Figure 6b) shows that the hydroxyl proton signal (H-1) near 4.8 ppm becomes broadened with reduced intensity. This phenomenon can be ascribed to enhanced intermolecular interactions after the introduction of piperazine groups, which renders hydroxyl groups situated in diverse local chemical environments. Such heterogeneity of chemical environments broadens the distribution of chemical shifts for hydroxyl protons, manifesting as peak broadening and apparent intensity attenuation. Meanwhile, the phenolic hydroxyl proton signal (H-6) at 8.52 ppm in Lig exhibits remarkably increased intensity in Pip-Lig. The Mannich reaction preferentially occurs at the aromatic hydrogen ortho to phenolic hydroxyl groups, altering the chemical environment and hydrogen-bonding state of phenolic hydroxyls. Partial dissociation from intensive hydrogen bonding leads to the increased peak intensity observed in the spectrum. Furthermore, new multiplet peaks emerge in the range of 2.5–3.5 ppm, which correspond to protons of methylene bridges (-CH_2_-N-, H-7) and piperazine rings (-N-CH_2_-CH_2_-N-, H-8) [39]. This directly verifies that piperazine-formaldehyde is successfully grafted onto the lignin skeleton via covalent bonds.

### 3.3. Analysis of the Flame-Retardant Properties of Blends

The flame-retardant properties of the blends were evaluated by vertical burning tests and LOI, with the results listed in Table 2. Sample#0, without any flame retardant, failed the UL-94 rating test and showed an LOI value of 17.8%, consistent with values reported for polyethylene in the literature [40,41]. Adding 30 wt% APP (Sample#1) increased the LOI to 23.0%, but still no flame-retardant rating was achieved and molten dripping occurred. This indicated that the flame-retardant efficiency of APP alone is relatively low. Introduction of Si-Cu-APP (Sample#2) raised the LOI to 25.0% and reached a V-2 rating, yet dripping was still observed, implying that the char layer formed was insufficiently compact with weak barrier performance. Sample#3 with Lig exhibited an LOI of only 19.5% with no rating, showing poor flame retardancy, which is related to the porous and loose char layer formed after pyrolysis. Sample#4 containing Pip-Lig gave an LOI of 22.0% and achieved V-2 rating but still experienced dripping, demonstrating that modified Lig alone cannot meet the demand for high-efficiency flame retardancy. When Si-Cu-APP and Pip-Lig were combined, the flame-retardant performance of the blend was significantly enhanced. In the Si-Cu-APP/Pip-Lig system, Sample#5 showed an LOI of 26.2%, reaching V-1 rating with no dripping. Sample#6 displayed the optimal flame-retardant effect, with an LOI as high as 28.5%, UL-94 V-0 rating, and no molten dripping, exhibiting excellent self-extinguishing ability and char layer protection. Notably, Sample #6 contained a lower phosphorus loading of Si-Cu-APP than Sample #5, yet achieves superior flame resistance. This distinctive phenomenon originates from the multi-element cooperative flame-retardant effect of P-N-Si-Cu triggered by the optimized proportion of the two components. The blending ratio of Sample #6 matches the reaction characteristics of the system. The low dosage of Si-Cu-APP enables silicon and copper modified moieties to fully exert catalytic charring effects. Meanwhile, nitrogen-rich modified lignin in Pip-Lig efficiently couples with phosphorus-containing components, facilitating the rapid formation of continuous, thermally stable intumescent char during matrix pyrolysis. This proportion avoids the drawbacks induced by high phosphorus loading, such as loose char structure and excessive volatilization of pyrolysis products. It greatly enhances the barrier shielding and flame suppression capacity of the char layer. As the proportion of Si-Cu-APP further decreased to ratios of 2:1 (Sample#7) and 1:1 (Sample#8), the LOI values dropped to 25.2% and 24.4% respectively. The rating fell back to V-2 with dripping observed, and the flame-retardant performance gradually declined. An unbalanced ratio disrupted the effectiveness of the cooperative fire-retardant mode of action. Compared with the Si-Cu-APP/Pip-Lig system, Sample#9 had an LOI of 26.5% and reached V-1 rating, while Sample#10 showed an LOI of 25.5% and V-2 rating. Both were inferior to Sample#6, confirming the advantage of the cooperative combination of Si-Cu-APP and Pip-Lig in improving the flame retardancy of HDPE.

### 3.4. Thermal Stability of Blends

**Figure 7:**
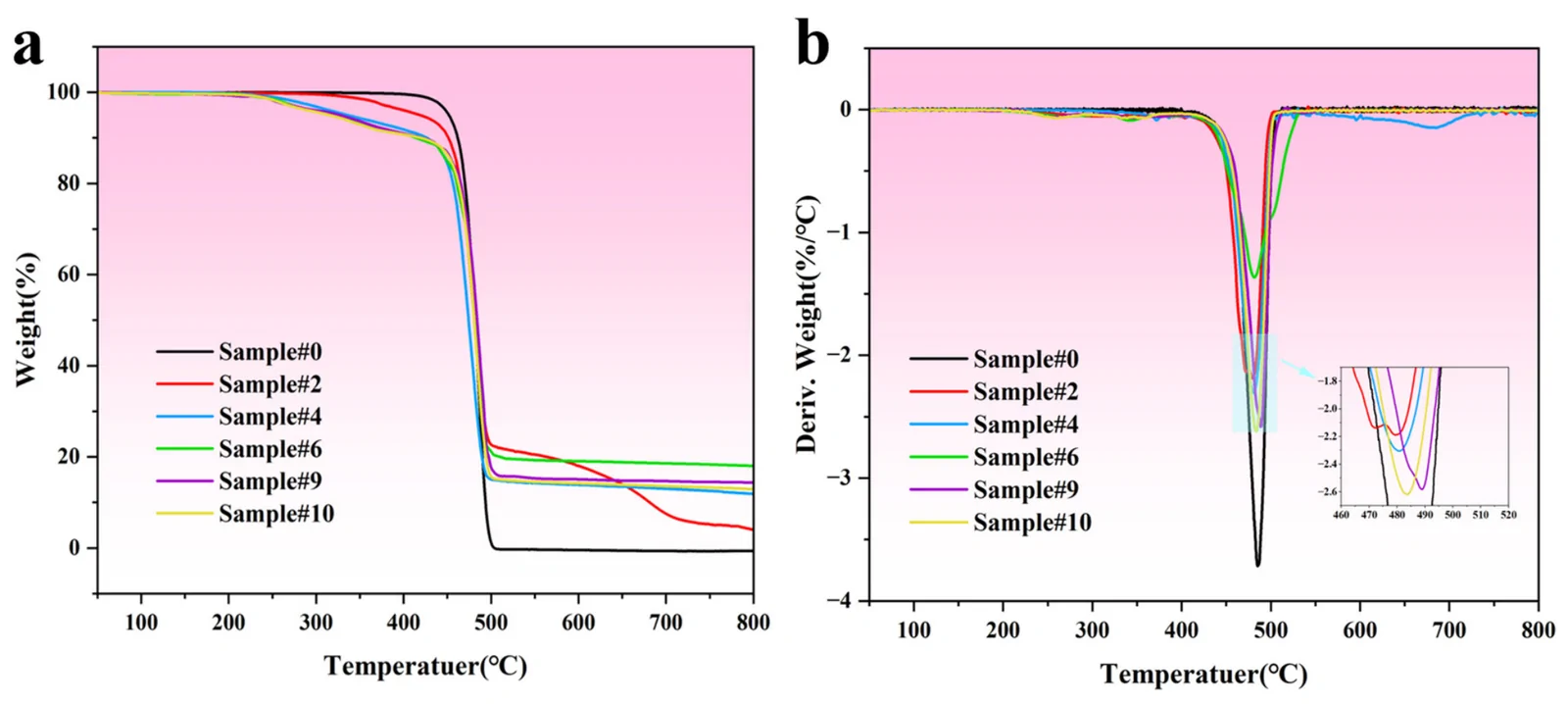
(**a**) TGA and (**b**) DTG curves of HDPE and its flame-retardant blends in N_2_ atmosphere.

Thermogravimetric analysis (TGA) was carried out under a nitrogen atmosphere to evaluate the thermal stability of the blends, with the results presented in Table 3 and Figure 7. HDPE (Sample#0) underwent severe one-step degradation in the temperature range of approximately 450–500 °C, leaving almost no char residue at 800 °C, indicating poor thermal stability [42]. After adding 30 wt% Si-Cu-APP (Sample#2), the initial decomposition temperature(T_5%_) of the blend decreased to 418.7 °C, while the char yield increased. This is because the phosphorus-containing components in Si-Cu-APP can catalyze char layer formation, thus improving the thermal stability of the material to some extent. When using Pip-Lig (Sample#4), the improvement in thermal stability was limited, and the char yield of 11.8 wt% remained relatively low. After combining Si-Cu-APP and Pip-Lig (Sample #6), the initial decomposition temperature (T_5%_) decreased to 315.5 °C. This phenomenon can be attributed to the catalytic interactions between Si-Cu-APP and Pip-Lig. During heating, phosphorus-containing species derived from Si-Cu-APP may generate acidic intermediates that promote dehydration and condensation reactions involving the nitrogen-containing functional groups and carbonaceous structures of Pip-Lig. These reactions may initiate mass loss at relatively low temperatures while facilitating the formation of carbonaceous precursors for subsequent char development. Meanwhile, the aromatic structure of lignin provides a carbon-rich framework, and the combined effects of phosphorus- and silicon-containing species may contribute to the formation and stabilization of condensed-phase residues. Therefore, the decrease in T_5%_ primarily reflects the initiation of decomposition and char-forming reactions at lower temperatures and does not necessarily indicate a deterioration in the overall flame-retardant performance. Consequently, the char yield at 800 °C reached the highest value of 18.1 wt%. Compared with Sample#2 and Sample#4, both T_5%_ and char yield of Sample#6 were significantly improved, indicating a strong cooperative effect between Si-Cu-APP and Pip-Lig. From the DTG curves, it can be seen that HDPE (Sample#0) shows a low T_max_ (maximum thermal decomposition peak), whereas the T_max_ of the blend system (e.g., Sample#6) shifts toward higher temperatures with a broader decomposition peak. This demonstrates that the cooperative effect effectively delays the thermal decomposition process of the material. Compared with Sample#9 and Sample#10, Sample#6 also exhibits obvious advantages in thermal stability. This further verifies that the cooperative effect between Pip-Lig and Si-Cu-APP effectively retards thermal decomposition and enhances the thermal stability of the blend.

It should be noted that the decrease in T_5%_ does not necessarily indicate a corresponding reduction in the processing temperature or long-term heat-resistant service temperature of the HDPE blend. T_5%_ represents the temperature at which a 5% mass loss occurs under the specific TGA conditions, and the initial mass loss may be associated with the decomposition of the flame-retardant components rather than extensive degradation of the HDPE matrix. In the present study, the mixing and hot-pressing temperatures were both set at 175 °C, which is approximately 140.5 °C lower than the T_5%_ of Sample#6 (315.5 °C). This substantial temperature difference suggests that the selected processing temperature is below the temperature at which 5% mass loss was observed under the TGA conditions, providing a favorable thermal margin for the preparation of the blend.

### 3.5. Cone Calorimetry Test

**Figure 8:**
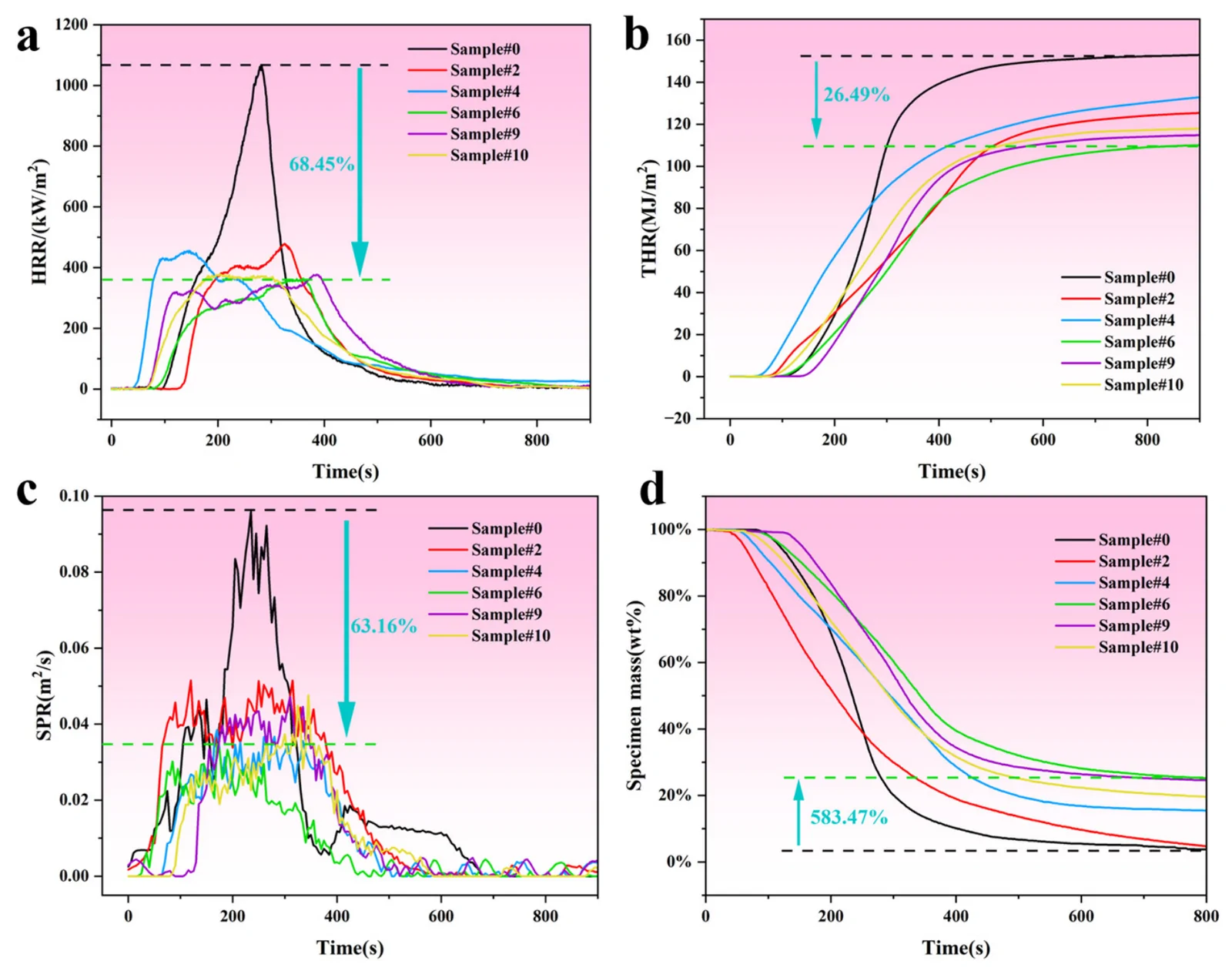
(**a**)HRR (**b**) THR (**c**) SPR and (**d**) Specimen mass curves of HDPE and its flame-retardant blends.

Cone calorimeter tests (CCT) were widely used to evaluate the fire hazard of polymer-matrix blends, as they can effectively simulate the combustion behavior of materials under real fire conditions (refer to Figure 8 and Table 4). As shown in Table 4, compared with flame-retardant-free Sample#0, the time to ignition (TTI) of each flame-retardant system varied differently. The TTI of Sample#2 was prolonged to 110 s, whereas those of Pip-Lig-containing samples (#4, #6, #9, #10) were shortened. This was mainly attributed to the different thermal decomposition pathways of Lig from HDPE, leading to early degradation of the system at lower temperatures [43]. In contrast, the high thermal stability of Si-Cu-APP delayed the ignition of the material. As can be seen from Figure 8a,b, HDPE (Sample#0) released heat rapidly after ignition, reaching PHRR of approximately 1066.2 kW·m^−2^ at about 277 s, with THR of about 153.04 MJ·m^−2^ and the lowest char residue. In comparison, Sample#2 showed PHRR of 488.74 kW·m^−2^ and THR of 127.19 MJ·m^−2^, representing reductions of 54.2% and 16.9% respectively compared with HDPE, while the char yield increased to 4.69 wt%. This indicates that phosphorus-containing components in Si-Cu-APP can catalyze char formation in the condensed phase, suppressing heat and flammable gas transfer to some extent [44]. Sample#4 exhibited PHRR of 455.97 kW·m^−2^, THR of 137.63 MJ·m^−2^, and an increased char yield of 15.48 wt%, showing good char-forming ability. When Si-Cu-APP and Pip-Lig were combined, the combustion behavior of the blend was significantly improved. Among them, Sample#6 achieved the optimal flame-retardant performance, with its PHRR reduced to 336.42 kW·m^−2^, THR to 112.5 MJ·m^−2^, and char yield greatly increased to 25.22 wt%, all being the best values among all samples. This result was superior to Sample#9 (PHRR 376.95 kW·m^−2^, THR 116.51 MJ·m^−2^, char yield 24.56 wt%) and Sample#10 (PHRR 378.16 kW·m^−2^, THR 119.22 MJ·m^−2^, char yield 19.55 wt%), fully demonstrating the significant cooperative flame-retardant effect between Si-Cu-APP and Pip-Lig. This synergy promoted the formation of a denser and more continuous protective char layer, which effectively blocked heat and mass transfer, thereby significantly reducing the combustion intensity of the material.

The peak CO_2_ production rate (Peak-CO_2_P) is an important parameter for evaluating combustion intensity. The Peak-CO_2_P of Sample#0 was as high as 0.491 g·s^−1^, while that of Sample#6 decreased to 0.202 g·s^−1^, the lowest among all samples. This trend is consistent with the lowest PHRR and THR values, indicating that the intensity of the combustion reaction was effectively suppressed. Smoke production rate (SPR) and TSP are key indicators for evaluating the smoke suppression performance of materials during combustion. As shown in Figure 8c and Table 4, Sample#6 exhibited a peak smoke production rate (PSPR) of 0.034 m^2^/s and TSP of 7.98 m^2^, both the lowest among all samples. Compared with HDPE (PSPR 0.095 m^2^/s, TSP 10.86 m^2^), these values decreased by 63.2% and 26.5% respectively. This indicates that the cooperative effect of Si-Cu-APP and Pip-Lig not only inhibited combustion heat release but also significantly reduced smoke emission. The fire performance index (FPI), calculated as the ratio of TTI to PHRR, characterizes the resistance of materials to fire flashover. An increased FPI value implies a lower flame spread rate and improved safety. Table 4 shows that Sample#6 exhibited the highest FPI value of 0.226 s·m^2^·kW^−1^. In contrast, the FPI of Sample#0 was only 0.072 s·m^2^·kW^−1^, further confirming the superior flame-retardant safety of Sample#6.

### 3.6. Condensed Phase Analysis

**Figure 9:**
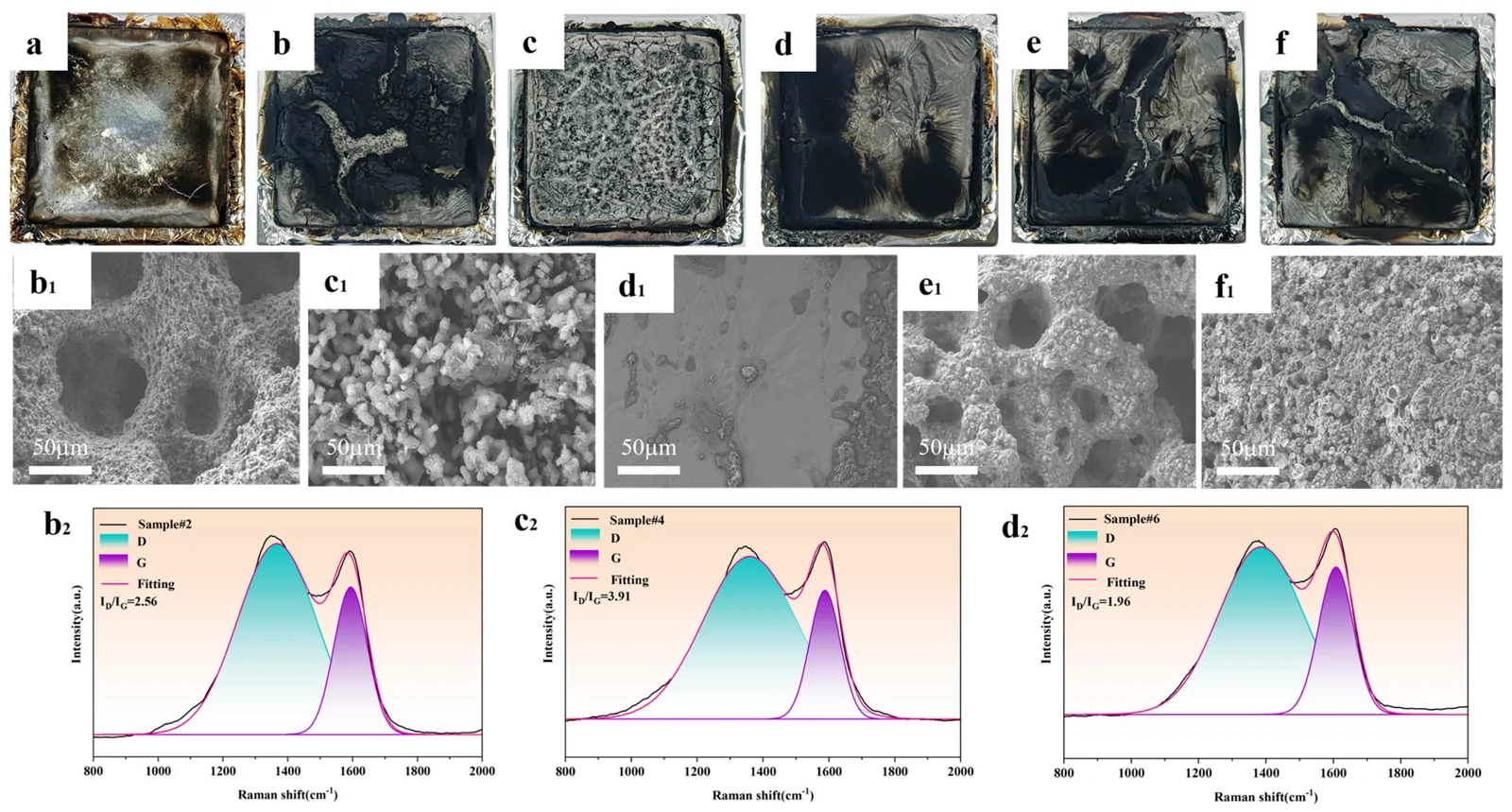
Digital photos of char residues for (**a**) Sample#0, (**b**) Sample#2, (**c**) Sample#4, (**d**) Sample#6, (**e**) Sample#9 and (**f**) Sample#10;SEM images of char residues for (**b_1_**) Sample#2, (**c_1_**) Sample#4, (**d_1_**) Sample#6, (**e_1_**) Sample#9 and (**f_1_**) Sample#10; Raman spectra of (**b_2_**) Sample#2, (**c_2_**) Sample#4 and (**d_2_**) Sample#6.

Systematic analysis of the char residues after cone calorimeter tests was conducted to elucidate the fire-retardant mode of action of the blends. As shown in Figure 9a, HDPE (Sample#0) left almost no char residue after combustion, indicating extremely high flammability. Sample#2 with Si-Cu-APP (Figure 9b) formed an intumescent char layer after combustion. However, obvious continuous microcracks appeared on its surface, suggesting that the structural integrity of the char layer needed improvement. The char residue of Sample#4 was relatively loose with numerous cracks. The cooperative effect of Si-Cu-APP and Pip-Lig (Sample#6, Figure 9d) resulted in a dense, smooth char layer surface with moderate intumescence, indicating that the introduction of lignin-based organic skeleton interpenetrating three-dimensional siloxane network and the catalytic charring effect of copper-containing species cooperatively improved the char layer quality. However, fine cracks also appeared in Sample#9 (Figure 9e) and Sample#10 (Figure 9f), revealing a remarkable bidirectional cooperative effect between Si-Cu-APP and Pip-Lig, without which the optimal char-forming effect could not be achieved. SEM images of the char residues supported this conclusion: the char layers of Sample#2 (Figure 9b_1_), Sample#4 (Figure 9c_1_), Sample#9 (Figure 9e_1_) and Sample#10 (Figure 9f_1_) exhibited obvious looseness and porosity at the microscopic level. This may be due to insufficient mechanical strength of the char layers, allowing volatile gases (such as NH_3_ and H_2_O) released during combustion to escape and form pores. In contrast, the char layer of Sample#6 (Figure 9d_1_) displayed a continuous and uniform microstructure, indicating superior compactness and mechanical strength over other formulations. Furthermore, Raman spectroscopic analysis of the char residues confirmed the above findings: the D/G band intensity ratios (I_D_/I_G_) of Sample#2 (Figure 9b_2_) and Sample#4 (Figure 9c_2_) were 2.56 and 3.91 respectively, indicating a high degree of structural disorder in the char layers. In comparison, Sample#6 (Figure 9d_2_) showed the lowest I_D_/I_G_ value of 1.96, reflecting a higher degree of graphitization. Higher graphitization enhances thermal stability and fire resistance, while effectively reducing heat release rate and CO_2_ emission.

### 3.7. Gas Phase Analysis

**Figure 10:**
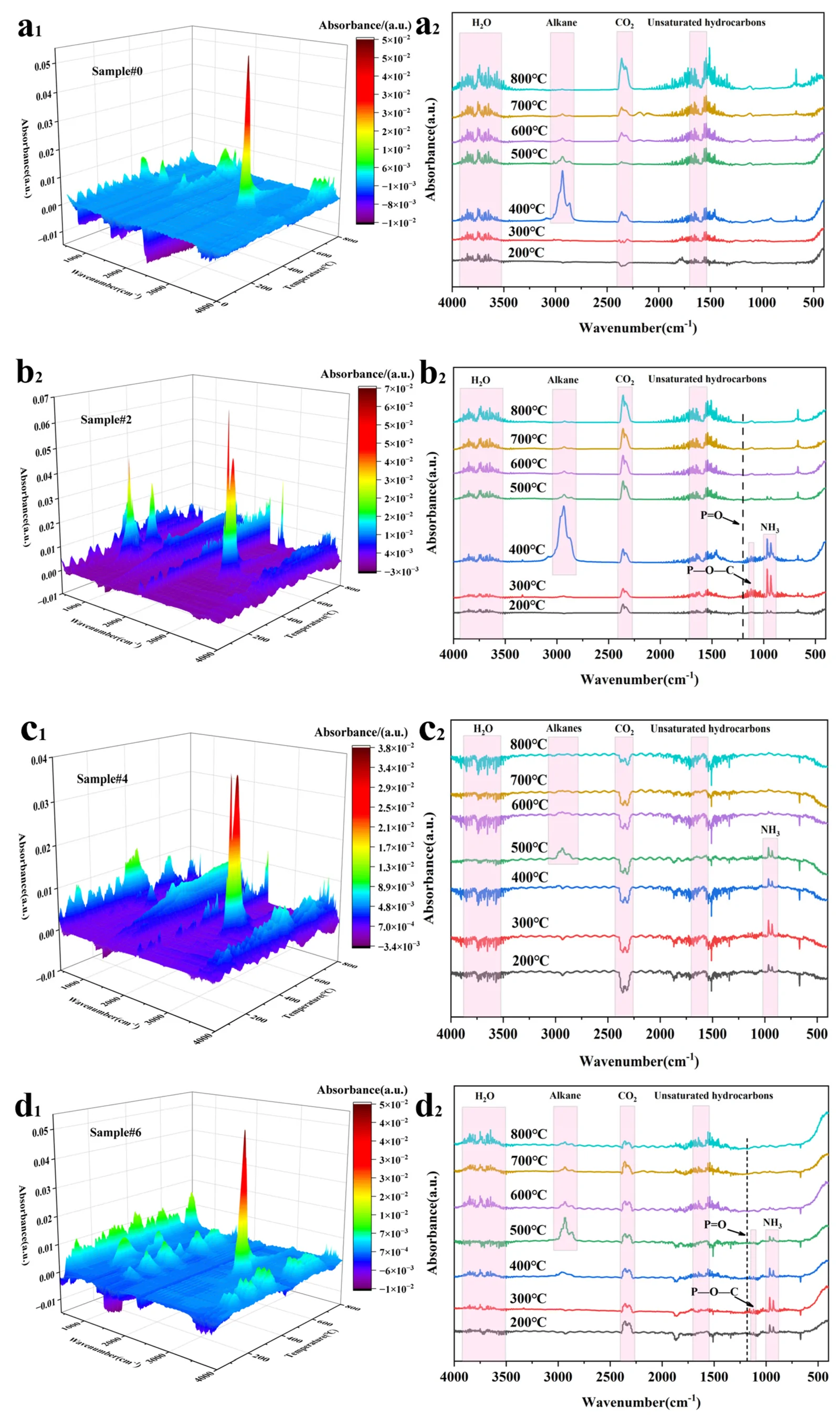
FTIR spectra of pyrolysis products of blends at different temperatures:(**a_1_**,**a_2_**) Sample#0; (**b_1_**,**b_2_**) Sample#2; (**c_1_**,**c_2_**) Sample#4; (**d_1_**,**d_2_**) Sample#6.

Thermogravimetry-Fourier transform infrared spectroscopy (TG-FTIR) was employed to investigate the thermal decomposition behavior and gaseous products of the blends. The results are presented in Figure 10 in the form of three-dimensional and two-dimensional spectra. For Sample#0 (Figure 10a_1_,a_2_), significant thermal degradation was observed at approximately 450 °C, accompanied by the release of large amounts of alkanes, alkenes, CO_2_ and H_2_O [45,46]. Upon the addition of Si-Cu-APP (Figure 10b_1_,b_2_), decomposition started at a lower temperature (250 °C), with the early release of alkanes and a small amount of NH_3_ [47]. This phenomenon was attributed to the rapid thermal decomposition of Si-Cu-APP, whose released phosphorus-containing groups (such as P=O and P–O–C) could catalyze the degradation of the HDPE matrix. At the same time, the decomposition of Si-Cu-APP facilitated the formation of a protective char layer, which blocked heat transfer and the escape of flammable volatiles, thereby significantly reducing the emission of alkanes and CO_2_. This observation is consistent with the decreased heat release rate in cone calorimeter tests. When Pip-Lig was used (Sample#4, Figure 10c_1_,c_2_), the release of NH_3_ during pyrolysis was significantly enhanced, while the release of alkanes, alkenes and CO_2_ was obviously reduced. The high nitrogen content in Pip-Lig was released as NH_3_ at high temperatures. As an inert gas, NH_3_ can effectively dilute flammable gases and lower the flammability of the combustion zone [48]. Meanwhile, the char-forming effect of modified Lig also suppressed the release of CO_2_ and other gases, improving the safety of the material. After combining Si-Cu-APP and Pip-Lig (Sample#6, Figure 10d_1_,d_2_), the release behavior of volatile products was further optimized. During pyrolysis, the release of NH_3_ from the blend system was more sustained and synchronized with the main degradation peak, which effectively diluted flammable gases such as alkanes and alkenes, inhibited the massive release of CO_2_, significantly reducing combustion intensity.

### 3.8. Fire-Retardant Mode of Action

**Figure 11:**
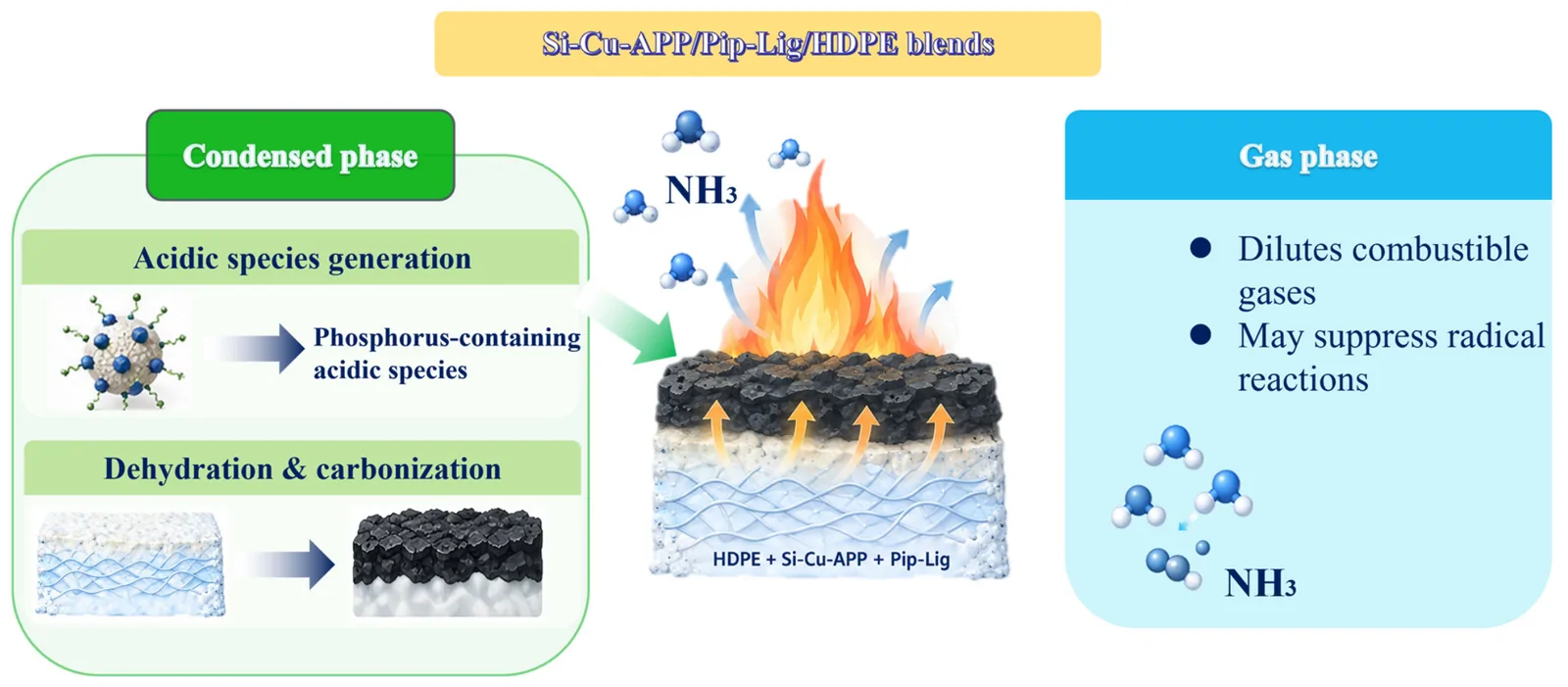
Fire-retardant mode of action of Sample#6 during combustion.

The analysis indicates that the flame-retardant mode of action of the HDPE blends is governed by the cooperative effects of the condensed phase and the gas phase, as illustrated in Figure 11. In the condensed phase, phosphorus-containing acidic species (such as polyphosphoric acid and phosphate esters) generated from the thermal decomposition of Si-Cu-APP catalyze the dehydration and carbonization of HDPE and Pip-Lig. Meanwhile, the incorporation of silicon- and copper-containing components may further influence the development and structural integrity of the carbonaceous residue. The aromatic structures of Pip-Lig provide an effective carbon source for char formation, while the cooperative interaction between Si-Cu-APP and Pip-Lig promotes the formation of a dense and stable protective char layer. Consequently, the residual char exhibits improved barrier properties, effectively inhibiting heat transfer, oxygen diffusion, and the release of combustible volatile products during combustion. In the gas phase, the thermal decomposition of Pip-Lig releases nonflammable gases, such as NH_3_, which dilute the concentration of combustible gases in the combustion zone and partially suppress free-radical chain reactions. Therefore, the cooperative effects of Si-Cu-APP and Pip-Lig in both the condensed and gas phases effectively reduce heat release and smoke generation, thereby significantly enhancing the flame-retardant and smoke-suppression performance of HDPE blends.

### 3.9. Water Resistance Analysis of Blends

#### 3.9.1. Surface Water Resistance

**Figure 12:**
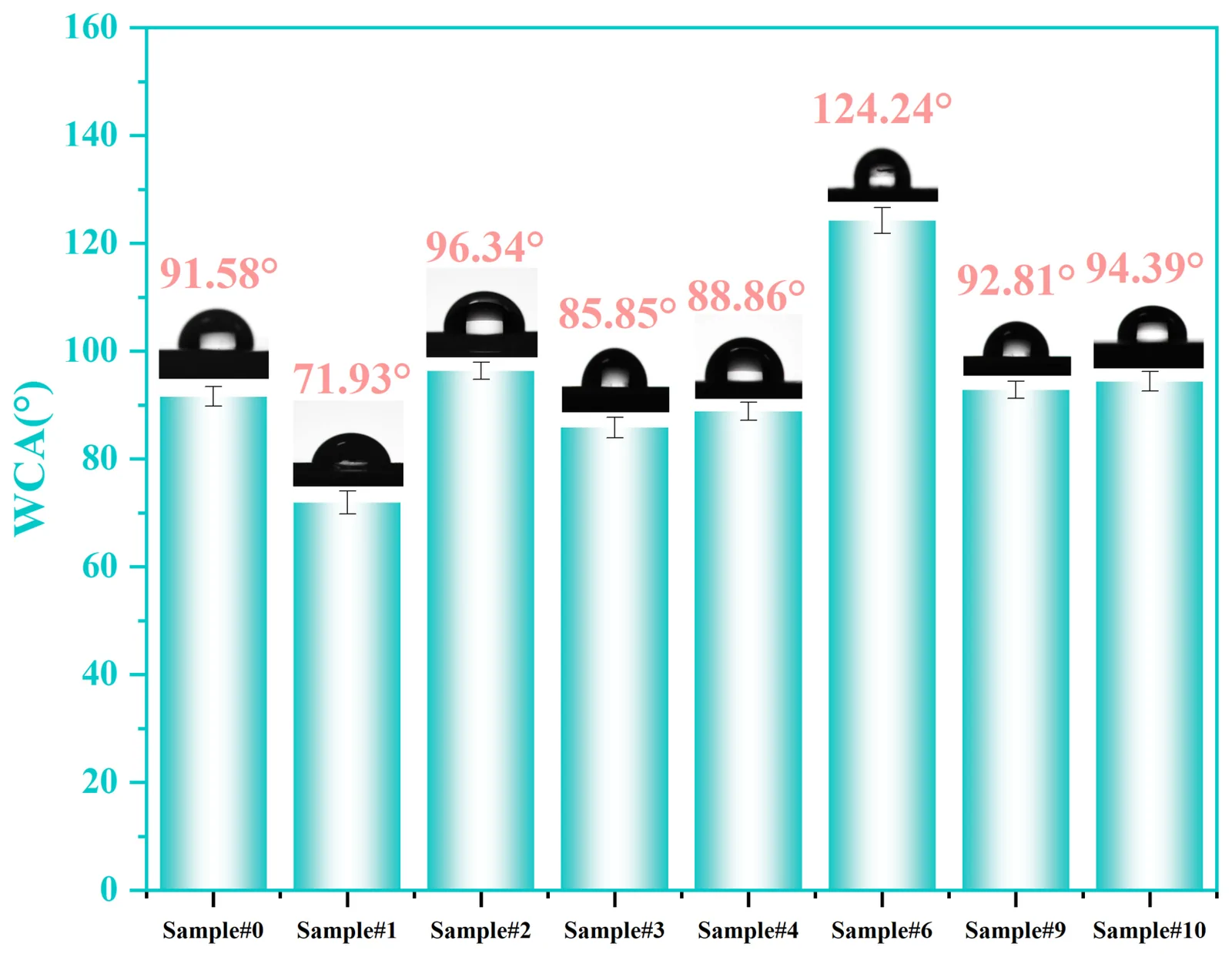
Water contact angle (WCA) images of Sample#0, Sample#1, Sample#2, Sample#3, Sample#4, Sample#6, Sample#9 and Sample#10.

The surface hydrophilicity and hydrophobicity of HDPE blends modified by different flame-retardant systems were systematically investigated via contact angle measurements, with the results shown in Figure 12. HDPE (Sample#0) exhibited a water contact angle of 91.58°, corresponding to weak hydrophobicity. After the addition of APP (Sample#1), the contact angle decreased significantly to 71.93°, rendering the surface hydrophilic. This was mainly attributed to the abundant polar phosphate and hydroxyl groups on the APP surface, which readily form hydrogen bonds with water molecules [49]. After the successive modification with Cu-MOF and KH550, the water contact angle of Si-Cu-APP (sample#2) increased to 96.34°, indicating a significant improvement in hydrophobicity. The introduction of Cu-MOF provides additional surface anchoring sites for the subsequent grafting of KH550, while the silane coupling agent forms a low-surface-energy silane layer on the APP surface, effectively shielding the hydrophilic groups and thereby improving the hydrophobicity of the modified APP [50]. The contact angle of Lig-containing Sample#3 was 85.85°. This is caused by abundant hydroxyl groups on the surface of lignin, which result in strong polarity and poor compatibility with the matrix, allowing water molecules to easily penetrate inward. In contrast, the contact angle of Pip-Lig-modified Sample#4 increased to 88.86°. This improvement in performance is attributed to the grafting of piperazine-formaldehyde onto Lig via the Mannich reaction. The hydrophobic piperazine rings grafted at the ortho-positions of the benzene rings shield the hydrogen-bonding interactions between hydroxyl groups through steric hindrance, reducing the surface energy and thereby enhancing the hydrophobicity of the material [51]. When Si-Cu-APP and Pip-Lig were combined at a mass ratio of 3:1 (Sample#6), the water contact angle reached 124.24°, the highest value among all samples, demonstrating high hydrophobicity. This improvement can be attributed to the cooperative effect between Si-Cu-APP and Pip-Lig. Following the successive modification with Cu-MOF and KH550, Si-Cu-APP possesses a lower surface energy, while the organic aromatic structure of Pip-Lig is considered to contribute to a less hydrophilic surface. Their cooperative effect is considered to reduce the surface affinity toward water, decreasing the wettability of the blend surface and thereby resulting in enhanced hydrophobicity. In comparison, the contact angles of APP/Pip-Lig (Sample#9, 92.81°) and Si-Cu-APP/Lig (Sample#10, 94.39°) were both lower than that of Sample#6, further confirming that the combination of Si-Cu-APP and Pip-Lig enabled the formation of the most stable hydrophobic surface structure. Such high hydrophobicity can effectively prevent moisture from penetrating into the blend interior, avoiding the failure of flame retardants caused by moisture absorption, significantly enhancing the flame-retardant durability of the material under humid environments.

#### 3.9.2. Resistance to Leaching Resistance

**Figure 13:**
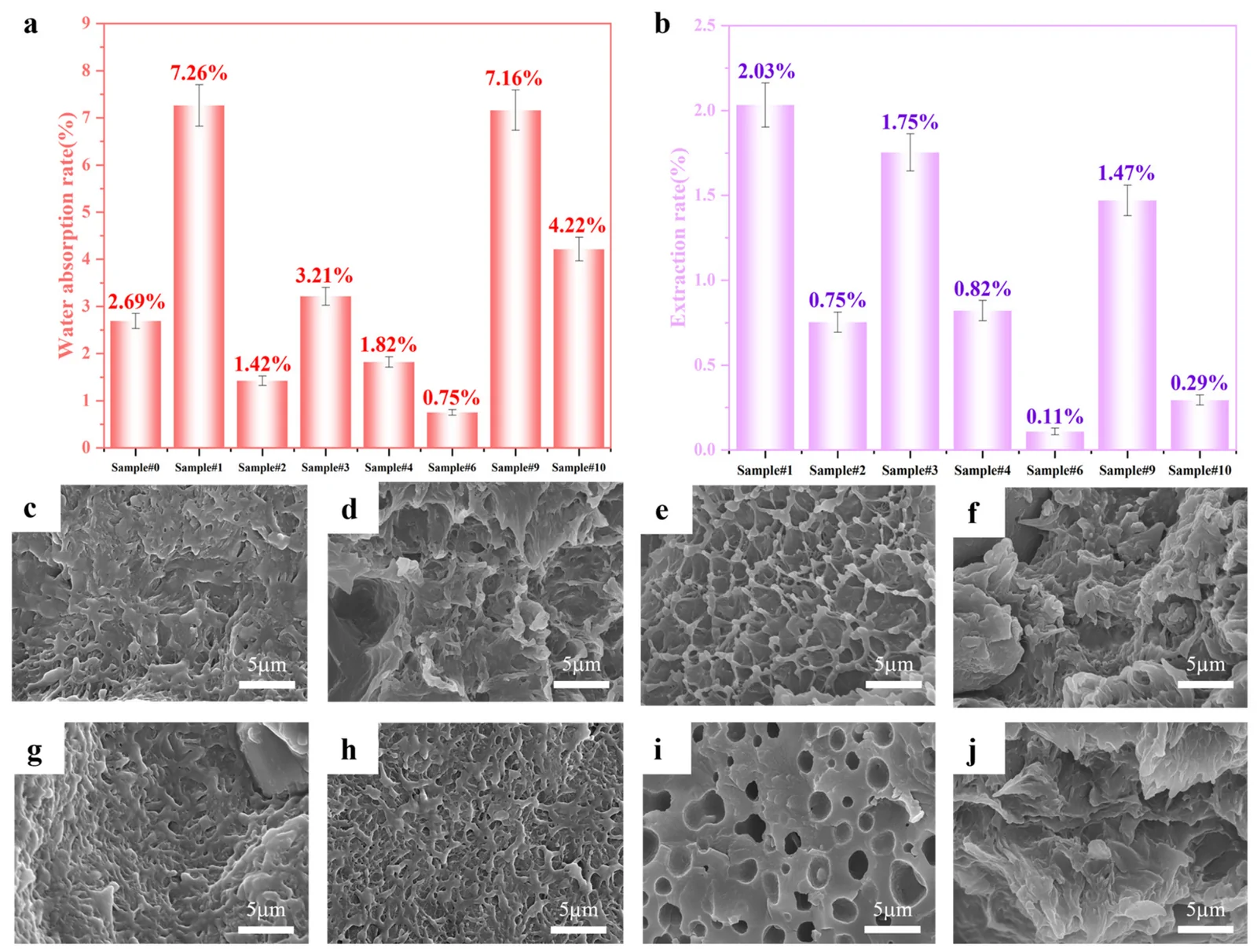
(**a**) Water absorption and (**b**) extraction rate plots of Sample#0, Sample#1, Sample#2, Sample#3, Sample#4, Sample#6, Sample#9 and Sample#10, as well as (**c**–**j**) SEM images of the fracture surfaces.

Polar flame retardant additive tended to leach out from HDPE under hot and humid conditions, which severely restricts its long-term flame-retardant performance. To investigate the water resistance of the blends, the extraction rate and water absorption of samples with different formulations were recorded during boiling migration tests, as shown in Figure 13. HDPE (Sample#0) showed a water absorption of 2.69%. After adding APP (Sample#1), the water absorption increased significantly to 7.26% and the extraction rate rose to 2.0%. This is because that APP has strong hydrophilicity. Moreover, its high polarity results in poor compatibility with the matrix, which makes it prone to absorbing moisture and exuding from the matrix in humid and hot environments [52]. After modification with KH550, the water absorption of Si-Cu-APP (Sample#2) decreased to 1.42% and the extraction rate to 0.75%, showing initial improvements in hydrophobicity and water resistance. Sample#3 containing Lig exhibited a water absorption of 3.2% and an extraction rate of 1.75%. This is due to the abundant hydroxyl groups on its surface. Lig exhibited strong polarity and poor compatibility with the matrix, making it prone to exuding in humid and hot environments. In contrast, Sample#4 with Pip-Lig showed a reduced water absorption of 1.82% and an extraction rate of 0.82%, demonstrating that the addition of modified Lig improves compatibility with the matrix, enhancing its hydrophobicity. When Si-Cu-APP and Pip-Lig were combined at a ratio of 3:1 (Sample#6), the blend achieved the best water resistance: a water absorption of only 0.7% and an extremely low extraction rate of 0.11%, far superior to all other formulations. The modification of APP with Cu-MOF and KH550 effectively reduces its hydrophilicity. While the cooperative interaction between Si-Cu-APP and Pip-Lig is considered to enhance the stability of the flame-retardant system during water exposure. Consequently, the contact between water molecules and the flame-retardant components is reduced, suppressing the migration and thereby leaching of the flame retardants during boiling-water treatment. In comparison, Sample#9 showed a water absorption of 7.16% and an extraction rate of 0.29%; Sample#10 exhibited a water absorption of 4.2% and an extraction rate of 1.47%. Both were far inferior to Sample#6 in water resistance, further confirming that the optimal ratio of Si-Cu-APP and Pip-Lig enables the most stable water-resistant structure.

In addition, SEM analysis of the blend fracture surfaces supported this conclusion. APP (Figure 13d) exhibits poor dispersion in the HDPE matrix, tending to form agglomerates and large voids. The dispersion of Si-Cu-APP (Figure 13e) is improved but still contains a small number of aggregates. Lig (Figure 13f) shows obvious agglomerates and large voids with poor interfacial compatibility, while Pip-Lig (Figure 13g) displays significantly improved dispersion with finer pores, though a few aggregates remain. Sample#9 presents a large number of circular macropores with severe defects, and Sample#10 exhibits a wrinkled fracture surface with local agglomeration and cracks, indicating the hydrophobic network is easily disrupted. In contrast, Sample#6 (Figure 13h) shows a smooth fracture surface with uniform and fine pores, demonstrating good dispersion of the flame retardant in the matrix and a stable hydrophobic network structure. The boiling-water migration test and dispersion analysis demonstrate that the cooperative flame-retardant system composed of Si-Cu-APP and Pip-Lig effectively enhances the water resistance of the HDPE blends. Following the successive modification of APP with Cu-MOF and KH550, the hydrophilicity of the flame retardant is effectively reduced. Meanwhile, the cooperative interaction between Si-Cu-APP and Pip-Lig is considered to promote a more stable dispersion of the flame-retardant system within the HDPE matrix. Consequently, the migration and leaching of the flame-retardant components during boiling-water treatment are effectively suppressed, leading to reduced water absorption and leaching ratio of the blends. As a result, the water resistance of the HDPE blends is significantly improved, which is beneficial for maintaining their flame-retardant performance after water exposure.

### 3.10. Comprehensive Performance Comparison of Vinyl-Based Polymers

**Figure 14:**
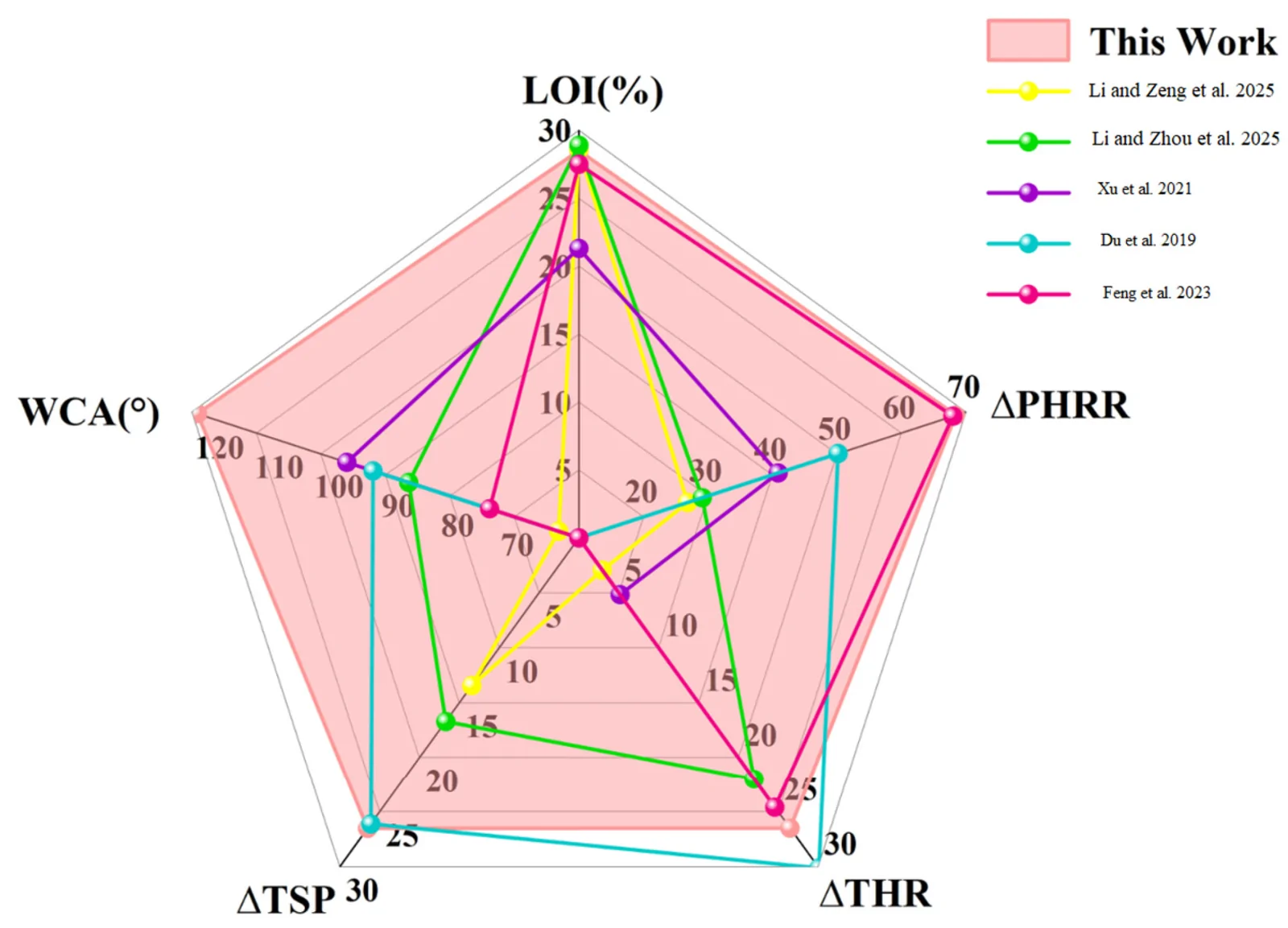
Comparison of flame-retardant and hydrophobic parameters in relevant references [53,54,55,56,57].

To comprehensively evaluate the comprehensive performance of this work, a multi-dimensional comparison was conducted with blend flame-retardant modification systems reported in recent literature that act on vinyl polymer matrices. Five core evaluation indicators were selected, including the LOI, WCA, peak heat release rate reduction (ΔPHRR), total heat release reduction (ΔTHR), and total smoke release reduction (ΔTSP). A radar chart was constructed for visual analysis (Figure 14).

In terms of flame retardancy: the ΔPHRR of this work reached 68.45%, which was significantly higher than the levels reported in references [53,54,55,56,57]. For ΔTHR and ΔTSP, this work achieved reductions of 26.49% and 26.52%, and the LOI was up to 28.5%. These results demonstrate the great potential of the hybrid system to suppress heat and smoke release under lab-scale combustion tests. In terms of hydrophobicity: the WCA of this work was as high as 124.24°, far exceeding that of the comparison systems. It exhibits favorable resistance against water penetration and flame-retardant migration under laboratory water-boiling tests, showing promising prospects for developing HDPE blends with improved water-resistant flame retardancy.

Unlike systems that excel in only a single performance, this work achieved a simultaneous improvement in high-efficiency flame retardancy and excellent hydrophobicity through the cooperative effect of dual modification with Si-Cu-APP and Pip-Lig.

## 4. Conclusions

This study constructs an organic-inorganic hybrid P-N-Si-Cu flame-retardant system based on Si-Cu-APP and Pip-Lig, and applies it to HDPE. The mass ratio of the two components is 3:1 with a total additive loading of 30 wt%. The blend achieves simultaneous improvements in flame retardancy and hydrophobicity. Compared with HDPE, the blend exhibits a LOI of 28.5% and a UL-94 V-0 vertical burning rating. Its PHRR, THR and TSP are reduced by 68.45%, 26.49% and 26.52%, respectively. The water contact angle increases from 91.58° to 124.24°, while the water absorption rate and extraction rate reach 0.75% and 0.11%, respectively. The above results demonstrate that the combination of Cu-MOF-loaded and KH550-modified APP with nitrogen-functionalized lignin can achieve synergistic improvements in the flame-retardant and water-resistant properties of HDPE blends under laboratory test conditions.

From an application perspective, this system provides a candidate formulation for HDPE products that require improved fire safety and moisture-resistance performance. For cable sheaths and other polymer products with high-performance requirements, subsequent formulation optimization should focus on reducing the total additive loading while maintaining desirable flame-retardant performance, and further evaluate melt-processing behavior, mechanical properties and electrical insulation performance.

Future research is suggested to focus on three aspects:

(i) Elucidate the independent action mechanism of each component through more in-depth characterization of combustion residues and thermal degradation products;

(ii) Establish an evaluation framework for the long-term stability of the flame-retardant system by assessing additive migration, water resistance, as well as mechanical and electrical properties after environmental aging;

(iii) Improve the scalable preparation capacity and practical application feasibility of this formulation by reducing filler loading, optimizing processing technologies, and conducting preliminary cost and environmental-impact assessments.

## Figures and Tables

**Table 1:** HDPE-based material ratios.

Sample	HDPE (wt%)	APP (wt%)	Si-Cu-APP (wt%)	Lig (wt%)	Pip-Lig (wt%)	APP/Si-Cu-APP: Lig/Pip-Lig
Sample#0	100					
Sample#1	70	30				
Sample#2	70		30			
Sample#3	70			30		
Sample#4	70				30	
Sample#5	70		24		6	4:1
Sample#6	70		22.5		7.5	3:1
Sample#7	70		20		10	2:1
Sample#8	70		15		15	1:1
Sample#9	70	22.5			7.5	3:1
Sample#10	70		22.5	7.5		3:1

**Table 2:** LOI and UL94 results for HDPE and its flame-retardant blends.

Sample	LOI (%)	UL-94 (3 mm)		Dripping
		t_1_ + t_2_(s)	Rating	
Sample#0	17.8 ± 0.11	>50	NR	Yes
Sample#1	23 ± 0.15	44.8	NR	Yes
Sample#2	25 ± 0.16	26.8	V-2	Yes
Sample#3	19.5 ± 0.12	>50	NR	Yes
Sample#4	22 ± 0.13	>50	V-2	Yes
Sample#5	26.2 ± 0.18	29.6	V-1	No
Sample#6	28.5 ± 0.2	12.5	V-0	No
Sample#7	25.2 ± 0.16	26.8	V-2	Yes
Sample#8	24.4 ± 0.15	34.3	V-2	Yes
Sample#9	26.5 ± 0.16	24.8	V-1	No
Sample#10	25.5 ± 0.15	26	V-2	Yes

**Table 3:** Thermal degradation data of HDPE and its flame-retardant blends.

Sample	T_5%_ (°C)	T_max_ (°C)	Char Residue (wt%)
Sample#0	451.7 ± 3.8	485.7 ± 1.8	0.5 ± 0.1
Sample#2	418.7 ± 3.2	479.7 ± 1.9	4.1 ± 0.4
Sample#4	335.7 ± 3.0	481.2 ± 1.7	11.8 ± 0.7
Sample#6	315.5 ± 2.8	480.2 ± 1.8	18.1 ± 0.9
Sample#9	326.8 ± 3.1	488.8 ± 2.0	14.3 ± 0.8
Sample#10	321.7 ± 2.9	484.3 ± 1.8	12.9 ± 0.7

**Table 4:** Cone results of HDPE and its flame-retardant blends.

Items	Sample#0	Sample#2	Sample#4	Sample#6	Sample#9	Sample#10
TTI (s)	77 ± 2	110 ± 4	39 ± 1	76 ± 2	51 ± 2	61 ± 2
Peak-CO_2_P (G·S^−1^)	0.491 ± 0.009	0.283 ± 0.005	0.236 ± 0.004	0.202 ± 0.003	0.221 ± 0.003	0.229 ± 0.003
THR (MJ·m^−2^)	153.04 ± 1.82	127.19 ± 1.65	137.63 ± 1.74	112.50 ± 1.53	116.51 ± 1.56	119.22 ± 1.61
PHRR (kW·m^−2^)	1066.2 ± 24.10	488.74 ± 13.21	455.97 ± 12.10	336.42 ± 10.56	376.95 ± 11.53	378.16 ± 11.97
PSPR (m^2^/s)	0.095 ± 0.002	0.053 ± 0.002	0.039 ± 0.001	0.034 ± 0.001	0.047 ± 0.002	0.048 ± 0.002
TSP (m^2^)	10.86 ± 0.25	18.37 ± 0.36	8.22 ± 0.21	7.98 ± 0.18	8.56 ± 0.23	9.21 ± 0.24
Char residue (wt%)	3.69 ± 0.17	4.69 ± 0.18	15.48 ± 0.25	25.22 ± 0.42	24.56 ± 0.39	19.55 ± 0.27
FPI (s·m^2^·kW^−1^)	0.072 ± 0.002	0.225 ± 0.004	0.086 ± 0.002	0.226 ± 0.004	0.135 ± 0.003	0.161 ± 0.003

## Data Availability

The data presented in this study are available on request from the corresponding author.
